# Amorphous Heterostructure Derived from Divalent Manganese Borate for Ultrastable and Ultrafast Aqueous Zinc Ion Storage

**DOI:** 10.1002/advs.202205794

**Published:** 2023-01-20

**Authors:** Xixian Li, Chenchen Ji, Jinke Shen, Jianze Feng, Hongyu Mi, Yongtai Xu, Fengjiao Guo, Xingbin Yan

**Affiliations:** ^1^ State Key Laboratory of Chemistry and Utilization of Carbon Based Energy Resources School of Chemical Engineering and Technology Xinjiang University Urumqi 830017 China; ^2^ State Key Laboratory of Fine Chemicals Dalian University of Technology Dalian 116024 China; ^3^ State Key Laboratory of Optoelectronic Materials and Technologies School of Materials Science and Engineering Sun Yat‐Sen University Guangzhou 510275 China

**Keywords:** aqueous Zn‐ion batteries, charge storage mechanism, high energy density, long lifespan, manganese borate

## Abstract

Aqueous zinc‐manganese (Zn–Mn) batteries have promising potential in large‐scale energy storage applications since they are highly safe, environment‐friendly, and low‐cost. However, the practicality of Mn‐based materials is plagued by their structural collapse and uncertain energy storage mechanism upon cycling. Herein, this work designs an amorphous manganese borate (a‐MnBO*_x_*) material via disordered coordination to alleviate the above issues and improve the electrochemical performance of Zn–Mn batteries. The unique physicochemical characteristic of a‐MnBO*_x_* enables the inner a‐MnBO*_x_* to serve as a robust framework in the initial energy storage process. Additionally, the amorphous manganese dioxide, amorphous Zn*_x_* MnO(OH)_2_, and Zn_4_SO_4_(OH)_6_·4H_2_O active components form on the surface of a‐MnBO*_x_* during the charge/discharge process. The detailed in situ/ex situ characterization demonstrates that the heterostructure of the inner a‐MnBO*_x_* and surface multicomponent phases endows two energy storage modes (Zn^2+^/H^+^ intercalation/deintercalation process and reversible conversion mechanism between the Zn*_x_* MnO(OH)_2_ and Zn_4_SO_4_(OH)_6_·4H_2_O) phases). Therefore, the obtained Zn//a‐MnBO*_x_* battery exhibits a high specific capacity of 360.4 mAh g^−1^, a high energy density of 484.2 Wh kg^−1^, and impressive cycling stability (97.0% capacity retention after 10 000 cycles). This finding on a‐MnBO*_x_* with a dual‐energy storage mechanism provides new opportunities for developing high‐performance aqueous Zn–Mn batteries.

## Graphical abstract

A conceptual amorphous manganese borate material for AZIBs is designed via a disordered coordination strategy. The unique physicochemical characteristic of a‐MnBO*_x_* can form the a‐MnO_2_, Zn*_x_* MnO(OH)_2_, and Zn_4_SO_4_(OH)_6_·4H_2_O phases, realizing multiple energy storage modes for enhancing the charge storage ability.

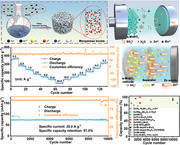

## 1 Introduction

Lithium‐ion energy storage devices with high energy density and capacity have dominated the commercial electrochemical energy storage market. However, their further large‐scale application has been limited by the resource shortage of crucial elements, environmental pollution, and the potential safety of commercial lithium batteries.^[^ ^1^ ^]^ Therefore, developing alternative energy storage systems with environmental friendliness, higher safety, and lower cost is urgently required. Aqueous zinc‐ion batteries (AZIBs) have aroused wide attention because of the high ionic conductivity of the aqueous electrolytes, low cost, abundant resources, and non‐toxicity.^[^ ^2^ ^]^ However, their rapid development is hindered by the limited host cathode materials and controversial mechanisms.^[^ ^3^ ^]^

Therefore, many efforts have been dedicated primarily to designing cathode materials for AZIBs, including vanadium‐based oxides, Prussian blue analogs (PBAs), organic compounds, and manganese‐based materials.^[^ ^3^, ^4^ ^]^ Although vanadium‐based cathodes feature high capacity, their high toxicity, high prices, and low operating voltage hinder their applications in AZIBs.^[^ ^3^, ^4^ ^]^ Furthermore, the PBAs and organic cathodes are often plagued by low discharge capacities or poor cycling stability.^[^ ^4b,c^ ^]^ In contrast, Mn‐based cathodes exhibit typical comprehensive advantages, such as environmental friendliness, non‐toxicity, and high output voltage (1.3–1.5 V), which may benefit the construction of AZIBs with essential properties of cost efficiency, high safety, and high energy densities.^[^ ^5^ ^]^

Till now, many works focused on the different crystal types (*α*, *β*, *γ*, *δ*, and *λ*) of Mn‐based materials,^[^ ^6^, ^7^, ^8^ ^]^ doping ions (N, Cu, La, and Ca),^[^ ^9^ ^]^ and their energy storage mechanisms^[^ ^10^ ^]^ to further optimize the electrochemical properties of Mn‐based systems. However, the investigation of the charge‐storage ability of amorphous Mn‐based materials in AZIBs has been paid little attention.^[^ ^11^ ^]^ Amorphous structures, bulk phases with disordered stacking of atoms, can buffer the stress and preserve its structural integrity during the ion intercalation/deintercalation process.^[^ ^11c^ ^]^ Simultaneously, the amorphous materials with many open pores are conducive to facilitating the diffusion of electrolyte ions. Additionally, the various homogeneous properties and short‐range ordering of amorphous structures enable low entropy energy when ions are embedded into the materials.^[^ ^12^ ^]^ Therefore, it is necessary to develop amorphous Mn‐based materials with boosted performance via feasible and effective strategies and to investigate their electrochemical properties and energy storage mechanism.

Herein, this work designed a conceptual amorphous manganese borate (denoted as a‐MnBO*_x_*) cathode material for AZIBs via disordered coordination between the divalent manganese ions (Mn^2+^) and the BO_3_^3−^ planar triangular or BO_4_^5−^ tetrahedral structures. The boracic ligands effectively maintain the divalent state of Mn^2+^ ions and enable the a‐MnBO*_x_* to possess a 3D robust porous structure with a large specific area (217 m^2^ g^−1^) and high electrochemical activity intrinsically. Moreover, it was demonstrated that the amorphous MnO_2_ (denoted as a‐MnO_2_), amorphous layered zinc‐manganese compound (denoted as Zn*_x_* MnO(OH)_2_), and zinc hydroxy sulfate (Zn_4_SO_4_(OH)_6_·4H_2_O, denoted as ZHS for simplicity) phases generate on the surface of a‐MnBO*_x_* during the charge/discharge process and act as active components for realizing the multiple energy storage modes (the insertion/extraction of ions and reversible conversion reactions). The above structural advantages endow the as‐built Zn//a‐MnBO*_x_* battery with a high reversible specific capacity of 360.4 mAh g^−1^ at 0.3 A g^−1^, outstanding rate performance of 77.7 mAh g^−1^ at a current rate of 20.0 A g^−1^, and long‐cycle life of 10 000 cycles at 20.0 A g^−1^ with an ultrahigh capacity retention of 97.0%. Moreover, the reaction mechanism of a‐MnBO*_x_* during the charge/discharge processes was elucidated by the in situ and ex situ characterization techniques and the density functional theory (DFT) calculations. Specifically, a‐MnBO*_x_* can form a heterogeneous structure with an inner part of a‐MnBO*_x_* and an outer part of multiphase components (a‐MnO_2_, Zn*_x_* MnO(OH)_2_, and ZHS phases). The inner a‐MnBO*_x_* acts as a “backbone” to maintain structural stability during the initial energy storage process. The outer layer of a‐MnO_2_ can provide stable active sites for the insertion/extraction of Zn^2+^/H^+^ ions. Meanwhile, the Zn*_x_* MnO(OH)_2_ phase can be reversibly converted into ZHS to enhance the charge storage ability further. This work will contribute to designing and developing amorphous Mn‐based materials with excellent electrochemical properties.

## 2 Results and Discussion

### 2.1 Morphology and Structural Characterizations of a‐MnBO*_x_*

**Figure 1:**
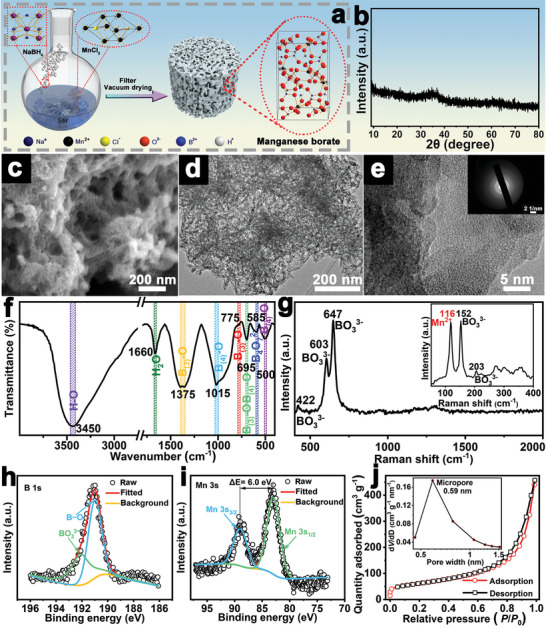
The schematic diagram for the synthesis of a‐MnBO*_x_* and the corresponding material characterizations. a) Schematic illustration of the synthesis process for a‐MnBO*_x_*. b) XRD pattern, c) representative SEM image, d) TEM image, e) HRTEM image, inset shows the corresponding SAED pattern, f) FTIR spectrum, g) Raman spectrum of a‐MnBO*_x_*. h) B 1s region and i) Mn 3s region of the XPS spectra. j) N_2_ adsorption/desorption isotherms, inset shows the corresponding pore size distribution.

The a‐MnBO*_x_* was synthesized via a one‐step coordination method starting with the inexpensive manganese chloride tetrahydrate (MnCl_2_·4H_2_O) and sodium borohydride (NaBH_4_), as shown in **Figure** **1**a and Figure S1, Supporting Information. The involved chemical reactions are as follows:^[^ ^13^ ^]^

(1) $${\mathrm{NaBH}}_{4}+2H_{2}O={\mathrm{NaBO}}_{2}+4H_{2}↑$$

(2) $${\mathrm{NaBO}}_{2}+2H_{2}O⇌H_{3}{\mathrm{BO}}_{3}+\mathrm{NaOH}$$

(3) $$4H_{3}{\mathrm{BO}}_{3}+2\mathrm{NaOH}={\mathrm{Na}}_{2}B_{4}O_{7}\cdot yH_{2}O+\left(7-y\right)H_{2}O$$

(4) $$\begin{array}{ccc} & & {\mathrm{Na}}_{2}B_{4}O_{7}\cdot yH_{2}O+{\mathrm{MnCl}}_{2}\to {\mathrm{MnB}}_{4}O_{7}\cdot yH_{2}O\left(a-{\mathrm{MnBO}}_{x}\right)\\ & & \;+2\mathrm{NaCl} \end{array}$$

It is worth noting that the B_4_O_7_^2–^ anion network is composed of alternate BO_3_^3−^ and BO_4_^5−^, which ensures the diversity of boron (B)‐based anion networks.^[^ ^13^ ^]^

The as‐prepared samples were analyzed by X‐ray powder diffraction (XRD). As shown in Figure 1b, except for a broad and weak peak around 35°, no other obvious characteristic diffraction peaks were detected in the XRD pattern of a‐MnBO*_x_*, revealing its amorphous structure. The morphology and microstructure of the samples were further investigated by scanning electron microscopy (SEM), transmission electron microscopy (TEM), and energy‐dispersive X‐ray (EDX) spectra. The a‐MnBO*_x_* sample consists of a rough surface and porous structure, which offers a sufficient exposed surface area for reversible electrochemical reactions and ion transfer (Figure 1c,d; Figure S2, Supporting Information). As shown in Figure 1e, the corresponding high‐resolution transmission electron microscopy (HRTEM) image revealed that no apparent crystal lattices were observed for the a‐MnBO*_x_* sample. The selected area electron diffraction (SAED) pattern displayed only a diffuse central blob without concentric diffraction rings (Figure 1e). Therefore, these characterizations demonstrated that a‐MnBO*_x_* possesses an amorphous structure. Additionally, the corresponding elemental mapping images revealed the uniform dispersion of Mn, B, and O atoms within the architecture (Figure S3, Supporting Information). Furthermore, based on thermogravimetric analysis (TGA), a‐MnBO*_x_* exhibits excellent thermal stability and does not even decompose at 400 °C (Figure S4, Supporting Information).

The composition of a‐MnBO*_x_* was further analyzed by Fourier‐transform infrared (FTIR) spectroscopy. As displayed in Figure 1f, five peaks located at 1375, 1015, 775, 695, and 500 cm^−1^ correspond to four vibrational modes of B—O bonds (in‐plane bending, out‐of‐plane bending, symmetric stretching, and asymmetric stretching vibrations), suggesting the existence of the basic building blocks of borates.^[^ ^14a,c^ ^]^ Additionally, it is worth noting that the peak at 585 cm^−1^ is caused by the symmetrical pulsating vibration of the B_4_O_7_^2−^ anion, which further elucidates the existence of B_4_O_7_^2−^ in a‐MnBO*_x_*.^[^ ^14c^ ^]^ As shown in Figure 1g, the Raman spectrum is characterized by intense bands at 603, 647, 150, and 116 cm^−1^. The peaks at 603 and 647 cm^−1^ are assigned to the asymmetric bending modes of the trigonal boron. The peaks located between 250 and 360 cm^−1^ are attributed to translational modes of BO_3_^3−^ groups and the translational motion of the oxygen atoms that is unbounded to boron atoms. The peak at 116 cm^−1^ results from the advective motion of Mn^2+^ ions, which further proves the presence of Mn^2+^ in a‐MnBO*_x_*.^[^ ^14^ ^]^

X‐ray photoelectron spectroscopy (XPS) was used to verify the coordination between Mn^2+^ and ligand in a‐MnBO*_x_*. The peaks at 653.8 and 642.1 eV in Mn 2p spectrum correspond to Mn^2+^ 2p_1/2_ and Mn^2+^ 2p_3/2_, respectively (Figure S5a–c, Supporting Information). As shown in Figure 1i, the energy separation between the Mn 3s doublet peaks is 6.0 eV. The valance state potential of a‐MnBO*_x_* is estimated to be +2.0.^[^ ^15^ ^]^ B 1s and O1s spectra suggest the existance of B—O bonds and BO_3_^3−^ in a‐MnBO*_x_* (Figure 1h,i; Figure S5d, Supporting Information).^[^ ^14a^ ^]^ The XPS elemental analysis also proved that the average molecular formula of a‐MnBO*_x_* is MnBO_2_·0.86H_2_O (Figure S6, Supporting Information). Furthermore, the N_2_ adsorption/desorption analysis confirmed that a‐MnBO*_x_* features a large specific surface area of 217 m^2^ g^−1^ and abundant micropores in the range of 0.5 to 0.7 nm (Figure 1j), significantly surpassing most of the reported Mn‐based materials.^[^ ^16^ ^]^ Therefore, the high specific surface area and pore distribution characteristics can provide abundant electrode/electrolyte contact interfaces and reduce ion diffusion paths, which benefits to accelerate the electrochemical kinetics of a‐MnBO*_x_*.

### 2.2 Electrochemical Performance of the Aqueous Zn//a‐MnBO*_x_* Battery

**Figure 2:**
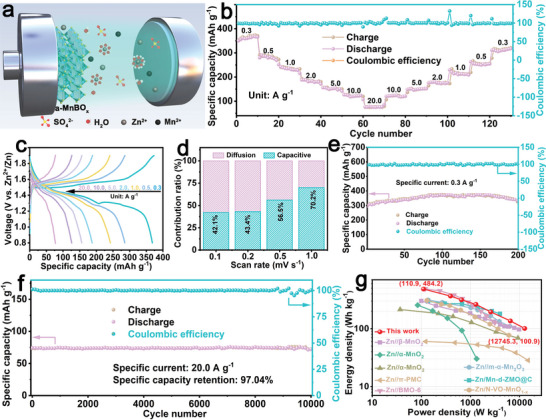
a) Schematic model of the aqueous Zn//a‐MnBO*_x_* battery. b) Rate capability for different current densities in the range of 0.3 to 20.0 A g^−1^. c) GCD profiles for different current densities in the range of 0.3 to 20.0 A g^−1^. d) Percentages of diffusion‐ and capacitive‐controlled contributions of the aqueous Zn//a‐MnBO*_x_* battery at different scanning rates. Long‐term cycling stability with the corresponding Coulombic efficiency of the aqueous Zn//a‐MnBO*_x_* battery at e) 0.3 A g^−1^ and f) 20.0 A g^−1^. g) Ragone plots of the aqueous Zn//a‐MnBO*_x_* battery and other reported Zn‐ion batteries.

As displayed in **Figure** **2**a, an aqueous Zn//a‐MnBO*_x_* battery was assembled using the a‐MnBO*_x_* cathode, Zn foil anode, glass fiber separator, and a mixed aqueous electrolyte (1 m ZnSO_4_ and 0.1 m MnSO_4_). A series of the related electrochemical tests were subsequently performed to evaluate the electrochemical performance of the as‐prepared a‐MnBO*_x_* samples. The rate capability of the aqueous Zn//a‐MnBO*_x_* battery is intuitively exhibited in Figure 2b. The as‐constructed aqueous Zn//a‐MnBO*_x_* battery delivers specific discharge capacities of 360.4, 285.6, 236.1, 188.3, 157.1, 125.9, and 77.7 mAh g^−1^ at current densities of 0.3, 0.5, 1.0, 2.0, 5.0, 10.0, and 20.0 A g^−1^, respectively. When the current density was returned to 0.3 A g^−1^, a specific discharge capacity of 317.3 mAh g^−1^ with a capacity retention of 88.0% was obtained. The obtain electrochemical performance is much better than that of the commercial *β*‐MnO_2_ material (Figure S7, Supporting Information). Thus, these results illustrate the high capacity and satisfactory rate property of a‐MnBO*_x_*. As shown in Figure 2c, the galvanostatic charge/discharge (GCD) curves of the Zn//a‐MnBO*_x_* battery show sloped and long flat plateaus at around 1.37 and 1.25 V, respectively. The increased voltage during discharge process (at the voltage of 1.30 V) in GCD curves should ascribe to the change in the charge‐storage mechanism at different stages, resembling the results of the previously reported MnO_2_.^[^ ^10^, ^17^, ^18^ ^]^

Furthermore, the contributions from capacitive processes and diffusion‐controlled redox reactions were calculated to explore the dynamics of the a‐MnBO*_x_* electrode.^[^ ^19^ ^]^ The calculated *b* value is shown in Figure S8a, Supporting Information. Previous works have reported that the *b* value is within 0.5–1.0. *b* = 0.5 implies a solid diffusion‐controlled process and *b* = 1.0 declares that the surface capacitive‐controlled is predominant.^[^ ^17^, ^19^ ^]^ The fitting results indicate that for all peaks, the fitted *b* values approach 0.5 (*b* values are 0.53 and 0.57 for redox peaks 1 and 2, respectively), which indicates solid diffusion‐controlled kinetics of a‐MnBO*_x_* during the charge/discharge process.^[^ ^17d^ ^]^ As shown in Figure 2d and Figure S8b, Supporting Information, the proportion of the capacitive‐controlled process is 42.1%, 43.4%, 56.5%, and 70.2% at 0.1, 0.2, 0.5, and 1.0 mV s^−1^, respectively. As shown in Figure S8c, Supporting Information, the electrochemical impedance spectroscopy (EIS) plot of the a‐MnBO*_x_* electrode composes of a small semicircle and a nearly straight line in the high and low‐frequency regions, respectively. The equivalent circuit diagram is shown in the inset of Figure S8c, Supporting Information. The fitting results of the EIS plot show that the charge transfer resistance (*R*_ct_) of the a‐MnBO*_x_* electrode is about 173 Ω. Furthermore, the high slope in the low‐frequency region indicates a smooth ion transfer property of the a‐MnBO*_x_* electrode. Furthermore, the self‐discharge test (Figure S8d, Supporting Information) of the aqueous Zn//a‐MnBO*_x_* battery was also conducted by resting it for 20 h after being fully charged to 1.9 V. Consequently, as shown in Figure S8d, Supporting Information, it has high‐capacity retention of 98.9% compared with the original charge capacity (366.0 mAh g^−1^), demonstrating an anti‐self‐discharge property.^[^ ^19d^ ^]^

Additionally, a long‐term cycling test of the aqueous Zn//a‐MnBO*_x_* device was performed to verify the advantages of the a‐MnBO*_x_* cathode. As displayed in Figure 2e, the specific capacity gradually increases at 0.3 A g^−1^ during cycling, which can be attributed to the gradual activation of a‐MnBO*_x_*.^[^ ^3d^ ^]^ The fluctuated coulombic efficiency in Figure 2e upon cycling may ascribe to Zn anode corrosion and zinc dendrites formation problems.^[^ ^2e^ ^]^ Remarkably, the specific capacity retention is close to 97.0% over 10 000 cycles under an ultra‐high current density of 20.0 A g^−1^, and the Coulombic efficiency per cycle is almost 100.0% (Figure 2f), affirming the excellent durability of the fabricated Zn//a‐MnBO*_x_* battery. Meanwhile, the Zn//a‐MnBO*_x_* battery also affords an ultra‐high energy density (*E*) of 484.2 Wh kg^−1^ at 110.9 W kg^−1^ and a high power density (*P*) of 12 745.3 W kg^−1^ at 100.9 Wh kg^−1^ based on the active mass of a‐MnBO*_x_* (Figure 2g). Furthermore, the maximal energy and power densities of the aqueous Zn//a‐MnBO*_x_* battery can outperform or be comparable to the values of most reported batteries with Mn‐based cathodes (Figure 2g; Table S3, Supporting Information), such as m‐*α*‐Mn_2_O_3_, Mn‐d‐ZMO@C, BMO‐6, *π*‐PMC, and N‐VO‐MnO_1−_ *_x_*.^[^ ^5^, ^20^, ^21^ ^]^

### 2.3 Structural and Morphological Evolution of a‐MnBO*_x_*

**Figure 3:**
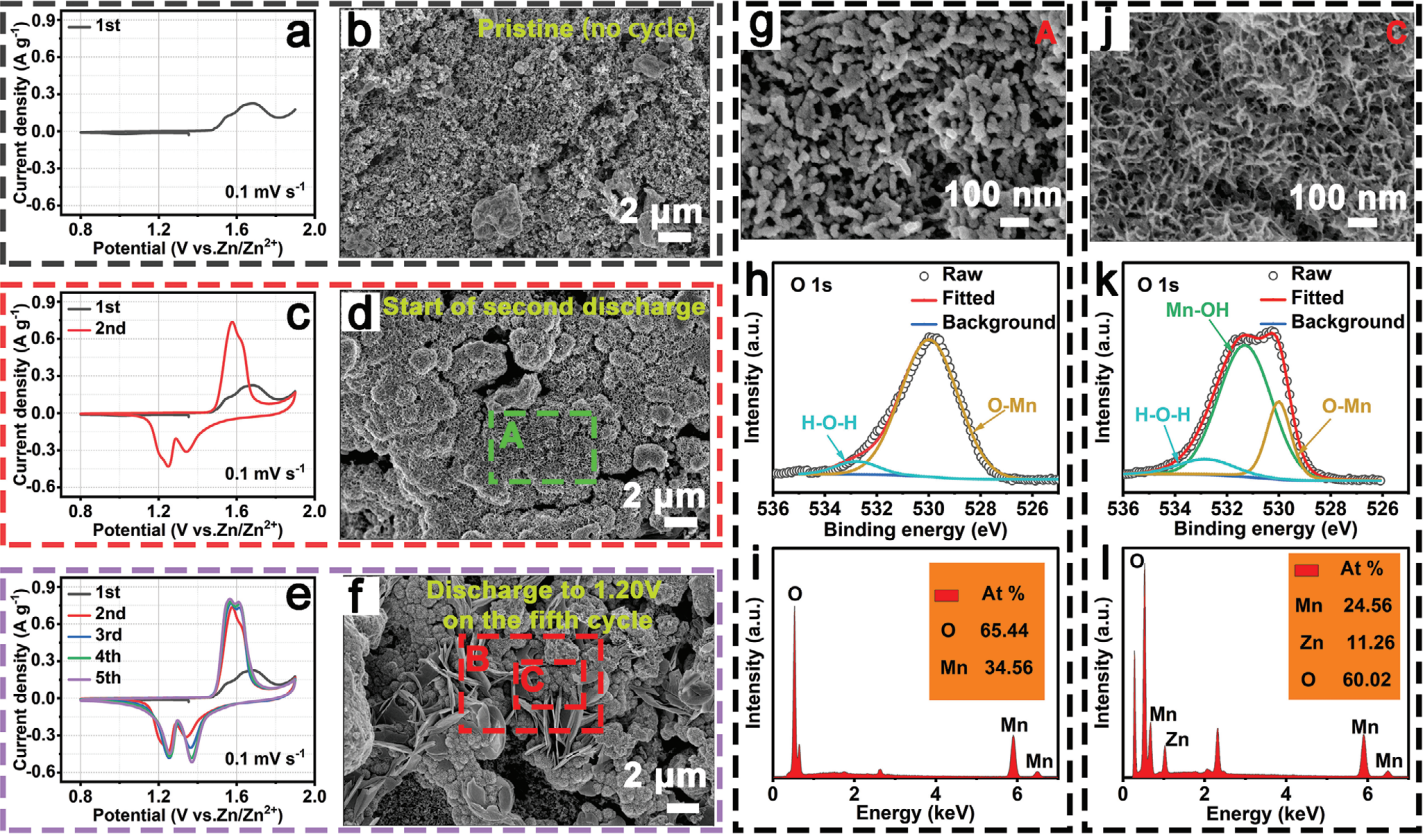
a) CV curve of the a‐MnBO*_x_* electrode at 0.1 mV s^−1^ for the first cycle. b) SEM image of the pristine a‐MnBO*_x_* electrode. c) CV curves of the a‐MnBO*_x_* electrode at a scan rate of 0.1 mV s^−1^ for the initial 2 cycles. d) SEM image of the a‐MnBO*_x_* electrode at the start of the second cycle. e) CV curves of the a‐MnBO*_x_* electrode at a scan rate of 0.1 mV s^−1^ for the initial 5 cycles. f) SEM image of the a‐MnBO*_x_* electrode at the discharge to 1.20 V on the fifth discharge cycle. g) SEM image of the enlarged part of region A in (d). h) The fine O 1s XPS spectrum of the a‐MnBO*_x_* electrode at the end of the first discharge cycle. i) EDS spectrum of the enlarged part at the end of the first charge cycle. j) SEM image of the enlarged part of region C in (f). k) The fine O 1s XPS spectrum at the discharge to 1.20 V of the fifth discharge. l) EDS spectrum of the enlarged part at the discharge to 1.20 V on the fifth discharge process.

Furthermore, SEM, TEM, XPS, and cyclic voltammetry (CV) measurements were performed to explore the evolution of the a‐MnBO*_x_* electrode. Previous works have observed no capacities at the initial discharge process in the GCD tests or no reduction peak during the initial cathodic scan from the CV curves for the divalent manganese oxide materials (e.g., MnO). This is ascribed to the non‐reactivity of Mn^2+^ that perhaps results from the non‐occurrence of charge storage reactions in Mn^2+^‐based material (e.g., the insertion reaction of cations or other conversion reactions) since Mn^2+^ ions with the lowest state are unable to store electrons by lowering their valence states.^[^ ^18d^ ^]^ A similar phenomenon was also observed in the prepared a‐MnBO*_x_* material. As shown in **Figure** **3**a, no reduction peak and negligible integral area were detected in the CV curve during the initial first cathodic scan process at 0.1 mV s^−1^, confirming that a‐MnBO*_x_* is incapable of storing charge before raising the valence of manganese ions. Meanwhile, the corresponding SEM images showed that the morphology of a‐MnBO*_x_* after the first cathodic scan process was similar to that of the pristine a‐MnBO*_x_* without morphology evolution (Figure 3b), further proving that no charge storage reactions occur in this process. However, obvious reduction peaks can be observed at 1.34 and 1.25 V versus Zn/Zn^2+^ during the cathodic scan in the second CV cycle (Figure 3c), which can be ascribed to the generation of the MnO_2_ phase (due to the reaction between the Mn^2+^ ions with the ZHS accompanied by continuously raising the valence of Mn after the anodic scan process) that enhances the discharge/charge capacities.^[^ ^18a^ ^,b]^ It is worth mentioning that the formed MnO_2_ phase would boost the charge storage ability by providing insertion/extraction sites for Zn^2+^/H^+^ ions. Additionally, the corresponding SEM image for the a‐MnBO*_x_* electrode after finishing the first cycle (completing the anodic scan process) showed the emergence of a large amount of rod‐like substances (Figure 3d). Furthermore, XPS and EDS measurements were performed to reveal the physicochemical properties of the newly emerged phases. The rod‐like structures in region A (Figure 3d,j; Figure S9a–c, Supporting Information) can be assigned to the amorphous‐MnO_2_ (a‐MnO_2_), as proven by XPS and energy dispersive spectroscopy (EDS) measurements.^[^ ^17^, ^18^ ^]^ The O1s fine spectrum after the first CV cycle displayed a peak located at 530.0 eV, which corresponds to the O—Mn bond of a‐MnO_2_ (Figure 3h and Figure S9d,e, Supporting Information).^[^ ^18^ ^]^ Meanwhile, two peaks at 654.0 and 642.4 eV in the high‐resolution XPS spectrum of Mn 2p designated to the binding energies of Mn 2p_1/2_ and Mn 2p_3/2_, respectively, corresponding to the Mn^4+^ species of a‐MnO_2_ phase (Figure S9d, Supporting Information).^[^ ^16^, ^22^ ^]^ EDS and mapping tests further elucidated the uniform distribution of Mn and O in region A (Figure S9b, Supporting Information). The ratio of Mn and O in EDS is close to 1:2, suggesting the formation of a‐MnO_2_ (Figure 3i; Figure S9d,e, Supporting Information). Therefore, the results collectively demonstrated that the rod‐like structures formed after the first CV cycle could be assigned to the amorphous MnO_2_. As expected, after the formation of the a‐MnO_2_ phase, distinct reduction peaks appearing at 1.37 and 1.26 V are observed with a high degree of overlap during the following cycles (e.g., the third, fourth, and fifth cycles) (Figure 3e). As shown in Figure 3f, discharge to 1.20 V on the fifth cycle, the a‐MnBO*_x_* electrode exhibits two morphologies, large micron‐sized flakes (region B) and small lamellar structures (region C, Figure 3f,g). The corresponding XRD results (Figure S10b, Supporting Information) demonstrated that the micron‐sized flakes in region B (Figure S10c,d, Supporting Information) are ascribed to the ZHS phase (PDF # 44–0673). The small lamellar structures in region C can be ascribed to the Zn*_x_* MnO(OH)_2_ phase (Figure S11a,b, Supporting Information).^[^ ^22^ ^]^ XPS and SEM‐EDS measurements were further performed to demonstrate these results further. As shown in Figure 3k, three peaks at 532.8, 531.3, and 530.0 eV in the O1s spectrum measured by XPS correspond to H—O—H, Mn—OH, and O—Mn, respectively.^[^ ^18^ ^]^ Additionally, it indicates the generation of MnO(OH)*_x_*‐containing substances during the discharge process. The EDS and mapping results further suggest the uniform distribution of Mn, O, and Zn in region C (Figure 3l; Figure S11e, Supporting Information). The non‐negligible content of the Zn element in the EDS results revealed that Zn^2+^ ions were involved in the energy storage process (Figure 3l; Figure S11e, Supporting Information). The above results reveal that the newly formed small lamellar structure in region C is Zn*_x_* MnO(OH)_2_.^[^ ^18a,b^ ^]^ Furthermore, XRD and HRTEM results (Figures S9c and S11b,c, Supporting Information) demonstrated that the a‐MnO_2_ and Zn*_x_* MnO(OH)_2_ phase possesses the amorphous structure. Additionally, Figure S12, Supporting Information, shows the SEM and the corresponding elemental mapping images of the Whatman GF/D (glass microfiber filters) separator from the Zn//a‐MnBO*_x_* battery at different charge/discharge processes. As shown in Figure S12, Supporting Information, Mn, O, S, and Zn are uniformly distributed among the whole GF/D separator at different charge‐discharge states (initial state, discharged to 0.8 V, and followed by being charged to 1.8 V). The corresponding SEM‐EDS elemental analysis of the a‐MnBO*_x_* electrode revealed that the relative content of the Mn element presents periodic changes at different charge‐discharge states. As shown in Figure S13, Supporting Information, the relative contents of O, S, and Zn elements remain unchanged at different charge and discharge processes. However, the relative content of the Mn element increases to 3.92 at% after being discharged to 0.80 V compared with the initial content of 0.91 at%. Moreover, the relative content of the Mn element recovers to 0.14 at% after being charged to 1.90 V. The obtained results proved that the surface of the a‐MnBO*_x_* material could partially dissolve into the electrolyte during the discharging process and Mn ion deposits on the electrode during the charging process. Additionally, photographs of the a‐MnBO*_x_* cathodes and Zn anodes at different cycles were provided to reveal the dissolution behavior of a‐MnBO*_x_*. As shown in Figure S14, Supporting Information, the a‐MnBO*_x_* cathodes gradually dissolve with an increase in the number of cycles, further proving the dissolution phenomenon of a‐MnBO*_x_* cathodes during the charge/discharge process. Hereto, the above results reveal that the outer part of the a‐MnBO*_x_* electrode can partially dissolve and release divalent manganese ions into the ZnSO_4_+MnSO_4_ electrolyte during the discharge process. Additionally, the Zn^2+^, SO_4_^2−^, and OH^−^ ions can gradually form the ZHS phase on the a‐MnBO*_x_* electrode during this process. Then, after the formation of ZHS, amorphous manganese dioxide and Zn*_x_* MnO(OH)_2_ are formed during the subsequent charging process. The electrochemical reaction process can be expressed as follows:^[^ ^18a,b^ ^]^

(5) $$\begin{array}{ccc} & & 3{\mathrm{MnB}}_{4}O_{7}\left(a-{\mathrm{MnBO}}_{x}\right)+6H^{+}+15H_{2}O\to 12H_{3}{\mathrm{BO}}_{3}\\ & & \;+3{\mathrm{Mn}}^{2+} \end{array}$$

(6) $$4{\mathrm{Zn}}^{2+}+{\mathrm{SO}}_{4}^{2-}+6{\mathrm{OH}}^{-}+4H_{2}O\to {\mathrm{Zn}}_{4}{\mathrm{SO}}_{4}{\left(\mathrm{OH}\right)}_{6}\cdot 4H_{2}O↓$$

(7) $$\begin{array}{ccc} & & 3{\mathrm{Mn}}^{2+}+2{\mathrm{Zn}}_{4}{\left(\mathrm{OH}\right)}_{6}{\mathrm{SO}}_{4}\cdot 4H_{2}O\to 3{\mathrm{MnO}}_{2}+8{\mathrm{Zn}}^{2+}+2{\mathrm{SO}}_{4}^{2-}\\ & & \;+14H_{2}O+6e^{-} \end{array}$$

(8) $$\begin{array}{ccc} & & {\mathrm{Zn}}_{4}{\mathrm{SO}}_{4}{\left(\mathrm{OH}\right)}_{6}\cdot 4H_{2}O+{\mathrm{Mn}}^{2+}\to {\mathrm{Zn}}_{x}\mathrm{MnO}{\left(\mathrm{OH}\right)}_{2}+4H^{+}\\ & & \;+{\mathrm{SO}}_{4}^{2-}+2e^{-} \end{array}$$

### 2.4 The Zn^2+^/H^+^ Intercalation/Deintercalation Mechanism of the Aqueous Zn//a‐MnBO*_x_* Battery

**Figure 4:**
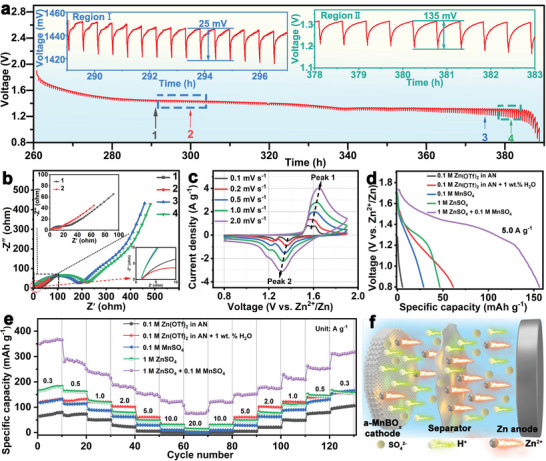
a) GITT profiles of the aqueous a‐MnBO*_x_* battery (at 50 mA g^−1^ for 120 s followed by a 0.5 h rest). b) EIS spectra at different depths of the discharge (marked by four arrows in (a)). The lower right inset shows the magnified region of the dotted circle part and the upper left inset shows magnified EIS at 1 and 2, respectively. c) CV curves of the aqueous Zn//a‐MnBO*_x_* battery at scan rates of 0.1, 0.2, 0.5, 1.0, and 2.0 mV s^−1^. d) The discharge profile of a‐MnBO*_x_* cathode at 5.0 A g^−1^ in different electrolytes. e) Rate capability at different current densities in the range of 0.3 to 20.0 A g^−1^ in the different electrolytes. f) Schematic illustration of the H^+^/Zn^2+^ charge‐storage mechanism.

The Zn^2+^/H^+^ intercalation/deintercalation process is widely recognized as the main electrochemical mechanism for the manganese dioxide material, but it is still unknown whether this process occurs for the a‐MnBO*_x_* material. In view to further reveal the electrochemical reaction mechanism, discharge galvanostatic intermittent titration technique (GITT) and EIS were performed. The reaction mechanism of the a‐MnBO*_x_* cathode in the above two plateaus (the sloped plateau at ≈1.37 V and the long flat plateau around 1.25 V) from the GCD curves (Figure 2c) was investigated using GITT and EIS. **Figure** **4**a shows the voltage response of the a‐MnBO*_x_* cathode from the GITT measurement, the total overvoltage in region II is 135 mV, which is almost six times higher than that in region I (25 mV) (inset in Figure 4a). However, the equilibrium plateau voltage for the electrochemical reaction in region II is lower than that in the region I. The large overvoltage in region II can be ascribed to the large voltage jump and slow ion diffusion. The voltage jump promptly after applying current in GITT is primarily resulted from the ohm and charge transfer resistances, while the gradual voltage change at the discharge stage attributes to the ion diffusion.^[^ ^23^ ^]^ The emergence of the two plateaus in the GITT curve with different overvoltages demonstrates two intercalation/deintercalation processes, which can be ascribed to the intercalation/de‐intercalation processes of Zn^2+^ and H^+^ ions. The obtained results are consistent with the reported works.^[^ ^23^ ^]^

Furthermore, significant differences in the charge transfer and diffusion process can be observed in the EIS plots, demonstrating different ion insertion processes. The EIS curves of the a‐MnBO*_x_* cathodes were obtained at four different stages in regions I and II, which are marked in Figure 4a, and the corresponding results are shown in Figure 4b. The inline‐image is partially enlarged from the corresponding area from Figure 4b. The EIS curves from different regions vary widely, and the two EIS spectrums in the same region are very similar. A comparative analysis of the EIS results shows that the ohmic resistances of regions I and II are the same. Significant differences are observed in Figure 4b in the charge transfer resistances and diffusion process of regions I and II, attributing to the different ion insertion processes. Considering only transferable Zn^2+^ and H^+^ ions in the electrolyte, the small impedance and overvoltage in stage I may result from the intercalation of H^+^ ions, and the larger impedance and overvoltage in stage II may be caused by the intercalation of Zn^2+^ ions because of their larger size.^[^ ^23b^ ^]^ A related XPS test was performed. It was observed that when the a‐MnBO*_x_* electrode was discharged to 1.40 V, no signals related to the Zn element were observed in Zn 2p regions (Figure S15a,b, Supporting Information). However, when the a‐MnBO*_x_* electrode continued to discharge to 1.30 V, plenty of Zn signals in Zn 2p spectra were observed. Therefore, these results further confirmed the reliability of the conclusion that regions I and II correspond to the intercalation processes of H^+^ and Zn^2+^ ions, respectively.^[^ ^23^ ^]^

According to the previous works, the reduction peak at 1.32 V and the peak at 1.23 V in the CV curve of the Mn‐based Zn ion batteries represent the intercalation of H^+^ and Zn^2+^, respectively.^[^ ^24^ ^]^ The CV curves at 0.1 to 2.0 mV s^−1^ are shown in Figure 4c. Apparently, a pair of reduction and oxidation peaks gradually weaken with increased scanning speed. The peak intensity of the insertion and extraction of H^+^ gradually strengthens at the high scanning rate.

Furthermore, a series of comparative experiments (e.g., 1 m ZnSO_4_, 0.1 m MnSO_4_, 0.1 m Zn(OTf)_2_/acetonitrile (denoted as AN, the solvent), and 0.1 m Zn(OTf)_2_/AN+1 wt% H_2_O) were performed to prove the intercalation/deintercalation mechanism of Zn^2+^/H^+^ ions in Zn//a‐MnBO*_x_* battery. The cells assembled with 1 m ZnSO_4_ + 0.1 m MnSO_4_ and 1 m ZnSO_4_ electrolytes displayed an obvious plateau at 1.30 V under small current densities (Figures S16–S18, Supporting Information). As shown in Figure 4d, when the current density increased to 5.0 A g^−1^, there were no obvious platform differentiators. However, the plateau at 1.30 V was not observed in the 0.1 m Zn(OTf)_2_/AN electrolyte at 0.3 or 5.0 A g^−1^. Moreover, a‐MnBO*_x_* provided a minimal capacity at any current density in the organic electrolyte, suggesting that H^+^ ions intercalation processes occur at 1.30 V plateau voltage. Furthermore, when 1 wt% H_2_O was added into the non‐aqueous electrolyte (0.1 m Zn(OTf)_2_/AN), the capacity showed a significant increase, confirming that H^+^ ions are involved in the energy storage process and dramatically contributed to the capacity (Figure 4e; Figures S19 and S20, Table S1, Supporting Information). The results further revealed that the synergistic insertion/extraction behaviors of Zn^2+^ and H^+^ ions occur during the energy storage processes for the a‐MnBO*_x_* electrode (Figure 4f).

### 2.5 Reversible Conversion Mechanism of the Aqueous Zn//a‐MnBO*_x_* Battery

**Figure 5:**
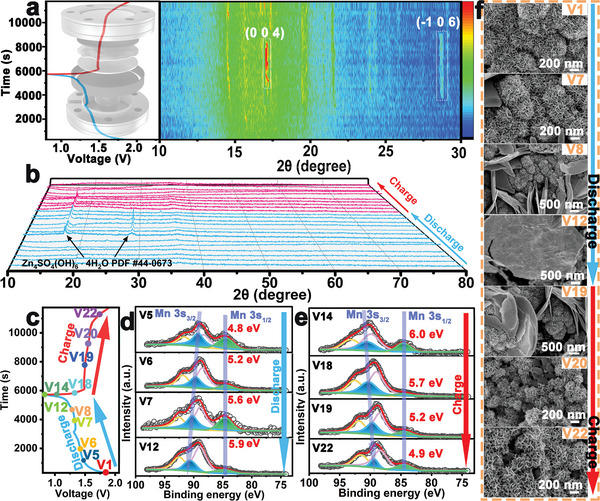
a) The contour plot shows the (004) and (−106) peaks in the in situ XRD pattern for different discharge–charge states (the left side shows the operando XRD measurement device). b) The ex situ XRD patterns of the a‐MnBO*_x_* cathode at different stages during a charge–discharge cycle. c) The galvanostatic charge and discharge profiles with the selected test points. The high‐resolution XPS spectra of Mn 3s regions for the a‐MnBO*_x_* cathode at d) different discharge states (at V5, V6, V7, and V12) and e) different charge states (at V14, V18, V19, and V22), respectively. f) FESEM images of the a‐MnBO*_x_* cathode at different charge–discharge states (at V1, V7, V8, V12, V19, V20, and V22).

Additionally, a reversible conversion reaction mechanism between ZHS and Zn*_x_* MnO(OH)_2_ was found for the a‐MnBO*_x_* electrode during the energy storage process. Therefore, as shown in **Figure** **5**a, an in situ XRD test based on the fifth charge/discharge circle was performed to reveal the crystal structure evolution of the electrode material. It was observed that the diffraction peaks of the (004) and (−106) crystallographic planes for the ZHS phase appeared and disappeared periodically. No other diffraction peaks were observed during the entire charge and discharge process in the *in‐situ* XRD pattern, which indicates the involvement of the periodic emergence of ZHS in the charge storage mechanism. As shown in Figure 5a, the characteristic diffraction peaks of ZHS appear after the Zn//a‐MnBO*_x_* battery is discharged to 1.30 V (vs Zn/Zn^2+^) and disappear after it is charged to 1.50 V (vs Zn/Zn^2+^), corroborating the good reversibility of ZHS. Furthermore, detail investigation were performed at various selected voltage (successively marked from V1 to V23) of the GCD curves for demonstrating the changes of the crystal structures, morphology, and valence state of a‐MnBO*_x_* cathode material (Figure S21, Supporting Information) during the charge/discharge process. As expected, similar results were also observed in the ex situ XRD patterns of the selected points (Figure 5b). Only the characteristic diffraction peaks of ZHS (at 17.1° and 25.8° in V7, V9, V12, V14, and V18) and no other peaks were observed, suggesting the energy storage process of the a‐MnBO*_x_* electrode contains the ZHS‐involved reversible conversion reactions. The HRTEM and SAED images of these points (Figure S22, Supporting Information) also demonstrated that the entire process exhibits a reversible conversion reaction between the ZHS and amorphous phases. As shown in Figure S10a, Supporting Information, a two‐electrode system using the Zn anode and a‐MnBO*_x_* cathode (on a stainless steel current collector) in a mixed aqueous electrolyte (1 m ZnSO_4_ and 0.1 m MnSO_4_) was employed to demonstrate the formation of ZHS further. As shown in Figure S10b, Supporting Information, after the a‐MnBO*_x_* cathode was charged to 1.9 V (vs Zn/Zn^2+^), it exhibited no other new characteristic peaks (except the characteristic peaks for stainless steel). However, the obvious typical characteristic peaks of ZHS (PDF # 44–0673) appeared after the cell was discharged to 0.8 V (vs Zn/Zn^2+^). Therefore, the obtained results demonstrated the formation of ZHS during the discharging process. Additionally, the SEM and TEM images in Figure S10c,d, Supporting Information, demonstrate the generation of large lamellae structures. The HRTEM image (Figure S10e, Supporting Information) displays three lattice fringes with the inter‐planar spacing of 0.52, 0.26, and 0.31 nm, which ascribed to the (004), (−106), and (3–12) crystal planes of ZHS, respectively. Moreover, the SAED pattern in Figure S10f, Supporting Information, verified the existence of ZHS. The diffraction rings can be indexed to the (004), (−106), and (3–12) crystal planes of ZHS. The obtained results are consistent with the XRD data, further proving the existence of ZHS during the discharging process.

Furthermore, XPS measurement was used to examine the specific chemical bonds and states at different discharge and charge stages during the energy storage process (Figure 5c–e; Figures S23 and S24, Supporting Information). As shown in the XPS spectra in Figure 5d,e, the splitting energies (Δ*E*) between the two Mn 3s peaks in Mn 3s spectra for the a‐MnBO*_x_* electrode increase from 4.8 eV (1.50 V, V5), 5.2 eV (1.40 V, V6), and 5.6 eV (1.30 V, V7), to 5.8 eV (0.80 V, V12) as the voltage discharged from the 1.50 to 0.80 V. Based on the linear relationship between the Mn chemical valence and Δ*E* value of Mn 3s, the determined average oxidation state of Mn is 3.6, 3.1, 2.6, and 2.3 at 1.50, 1.40, 1.30, and 0.80 V versus Zn/Zn^2+^ at these points, respectively.^[^ ^25^, ^26^ ^]^ Moreover, the O 1s spectra collected from the a‐MnBO*_x_* cathode can be fitted into two characteristic peaks around 531.3 and 529.8 eV, which associate with the Mn—OH and O—Mn bonds, respectively (Figure S24, Supporting Information).^[^ ^18^ ^]^ The average chemical composition at various charge/discharge states were obtained by combining the fine spectra of O 1s and Zn 2p. As shown in Mn 3s and Zn 2p spectra of Figure 5d,e and Figure S24, Supporting Information, it can be determined that the average chemical formulae at V5 (1.50 V) and V6 (1.40 V) are Zn_0.36_MnO(OH)_2.36_ and Zn_1.42_MnO(OH)_3.8_, respectively. Similarly, when the a‐MnBO*_x_* electrode charges to V14 (1.0 V), Δ*E* between the two Mn 3s peaks in Mn 3s spectra is 6.0 eV, which decreases to 5.7, 5.2, and 4.9 eV as the a‐MnBO*_x_* electrode further charges to V18 (1.40 V), V19 (1.50 V), and V22 (1.80 V), respectively. Therefore, the average valence of Mn is determined to be 2.1, 2.4, 3.1, and 3.5 at 1.0, 1.40, 1.50, and 1.80 V versus Zn/Zn^2+^, respectively. Based on the test results, the average chemical formula of the a‐MnBO*_x_* electrode in V19 and V22 can be ascribed to Zn_1.05_MnO(OH)_3.12_ and Zn_0.66_MnO(OH)_2.74_, respectively. The in situ and ex situ XRD patterns at the selected voltage (V1–V6, and V19–V23) indicated that Zn*_x_* MnO(OH)_2_ possesses an amorphous structure. Thus, these results further demonstrated periodic valence changes of Mn ions at different charge/discharge states and repetitive formation of Zn*_x_* MnO(OH)_2_ phase during the charging process. Furthermore, the periodic emergence of ZHS in the discharge process and the formation of Zn*_x_* MnO(OH)_2_ phase at the charge state corroborate the reversible conversion mechanism between the two phases.

Furthermore, the morphological evolution at different charge/discharge states was investigated by the FESEM, TEM, HRTEM, and SAED measurements. As shown in Figure 5f and Figure S25, Supporting Information, the ZHS phase with the micron‐sized flakes gradually emerged on the surface of the electrode after discharging to 1.3 V versus Zn/Zn^2+^ and completely transformed into small lamellar structures (corresponding to the Zn*_x_* MnO(OH)_2_ phase) after subsequent charging to 1.6 V. Moreover, the HRTEM images shown in Figure S11b, Supporting Information, verified that the Zn*_x_* MnO(OH)_2_ phase (honeycomb‐like structure assembled with small lamellar structures) completely disappeared. Only micron‐sized flakes of the ZHS phase and some irregular particles were observed after fully discharged to 0.8 V for 2 h, revealing that the Zn*_x_* MnO(OH)_2_ phase was gradually dissolved during discharging process. These results demonstrated a reversible dissolution‐deposition reaction of Zn*_x_* MnO(OH)_2_ at different energy storage processes. The above results are consistent with the XRD and XPS data, proving the reversible formation‐disappearance cycle between ZHS and Zn*_x_* MnO(OH)_2_. Thus, based on the test results, the reversible conversion mechanism can be described as follows: the ZHS phase formed during the discharging process. Then the Mn^2+^ ions interact with the generated ZHS phase via a redox reaction to form the Zn*_x_* MnO(OH)_2_ phase and are deposited on the surface of the electrode during the charge process (start from 1.5 V vs Zn/Zn^2+^). A subsequent increased manganese valence state and the variation in the constitute of the Zn*_x_* MnO(OH)_2_ phase occur upon the following charging process. During the following discharge process, ZHS re‐formed coupled with the dissolution of the Zn*_x_* MnO(OH)_2_ phase.^[^ ^18a,b^ ^]^ The obtained results revealed a ZSH‐assisted reversible conversion mechanism between the ZHS and Zn*_x_* MnO(OH)_2_ phase via a deposition‐dissolution behavior.

**Figure 6:**
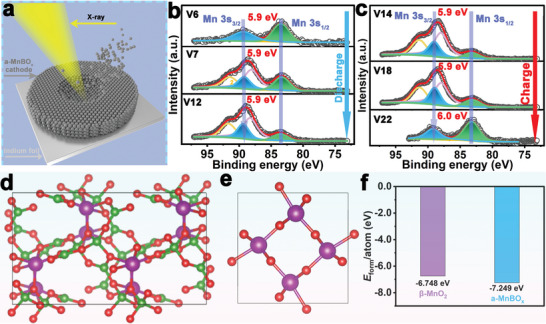
a) Schematic diagram of the etched‐XPS model for the a‐MnBO*_x_* electrode. High‐resolution Mn 3s XPS spectra after etching the a‐MnBO*_x_* electrode at b) different discharge states (V6, V7, and V12) and c) different charge states (V14, V18, and V22), respectively. The calculation models of the formation energy for d) a‐MnBO*_x_* and e) *β*‐MnO_2_, respectively. f) The formation energies of a‐MnBO*_x_* and *β*‐MnO_2_ from the DFT calculations.

Furthermore, the etched XPS measurement was performed to reveal the changes in the internal a‐MnBO*_x_* during the energy storage process, and the corresponding etching model is shown in **Figure** **6**a. As shown in Figure 6b,c, the peaks in the etched Mn 3s spectrum of the a‐MnBO*_x_* electrode after the initial five charge/discharge cycle remain unchanged compared with the periodic valence change in the non‐etched Mn 3s XPS fine spectrum (Figure 5d,e). This indicates that the inner part of a‐MnBO*_x_* does not undergo the valence state transformation for the Mn ions in the initial five charge/discharge cycles. The above results indicated that the charge storage processes occur primarily in the outer components (the newly generated a‐MnO_2_, Zn*_x_* MnO(OH)_2_, and ZHS) during the initial energy storage process. Similar results can also be drawn from the Mn 2p fine spectrum in Figure S26, Supporting Information, and the Mn element maintains its chemical state of +2 valence during the charge‐discharge process. This further illustrates the stable framework of inner a‐MnBO*_x_* in the initial electrochemical reaction process. Additionally, as shown in Figure S14, Supporting Information, the phenomenon of the a‐MnBO*_x_* cathode was gradually dissolved in the charge–discharge process indicates that the surface redox reactions progressively transitioned from the outermost layer to the inner part for the a‐MnBO*_x_* cathode material.

Additionally, DFT calculations were further used to verify the structural advantages of a‐MnBO*_x_*. The *β*‐MnO_2_ (composed of MnO_6_ octahedral basic units) was used for comparison to highlight the advantages of a‐MnBO*_x_* (Figure 6e). As shown in Figure 6f, the *β*‐MnO_2_ has a formation energy of −6.748 eV. However, more negative formation energy of −7.249 eV was found for a‐MnBO*_x_* (with the BO_3_^3−^ triangular and BO_4_^5−^ tetrahedral structures as the basic units) (Figure 6d,f). The DFT calculations further demonstrated that the a‐MnBO*_x_* possesses a more stable structure.^[^ ^27^ ^]^

Hereto, the above results revealed that three phases of a‐MnO_2_, Zn*_x_* MnO(OH)_2_, and ZHS form on the surface of a‐MnBO*_x_* to form a heterostructure as the charging and discharging process progresses and act as the active components for the energy storage, which exhibit different reaction mechanisms during the entire charge‐discharge process. Meanwhile, the inner part of a‐MnBO*_x_* can serve as a robust framework during the initial energy storage process. The physicochemical characterizations have manifested that two energy storage modes are involved in the charge–discharge process of the a‐MnBO*_x_* cathode material, which can be ascribed as follows: 1) Intercalation/deintercalation of H^+^ and Zn^2+^ ions in a‐MnO_2_ phase (formed on the surface of the a‐MnBO*_x_* cathode during the charging process). 2) A reversible conversion mechanism between ZHS and Zn*_x_* MnO(OH)_2_. The ZHS can interact with the Mn^2+^ ions via a redox reaction to form the Zn*_x_* MnO(OH)_2_ phase during the charging process. ZHS was re‐formed coupled with the dissolution of the Zn*_x_* MnO(OH)_2_ phase during the discharge process. From the above discussions, it was speculated that the main electrochemical storage reactions for the Zn//a‐MnBO*_x_* battery might be expressed as follows (the detailed reaction in Table S2, Supporting Information):

Cathode:

(9) $$H_{2}O↔H^{+}+{\mathrm{OH}}^{-}$$

(10) $$6{\mathrm{OH}}^{-}+{\mathrm{SO}}_{4}^{2-}+4{\mathrm{Zn}}^{2+}+4H_{2}O↔{\mathrm{Zn}}_{4}{\mathrm{SO}}_{4}{\left(\mathrm{OH}\right)}_{6}\cdot 4H_{2}O$$

(11) $$\begin{array}{ccc} & & {\mathrm{Zn}}_{4}{\mathrm{SO}}_{4}{\left(\mathrm{OH}\right)}_{4}\cdot 4H_{2}O+{\mathrm{Mn}}^{2+}\to {\mathrm{Zn}}_{x}\mathrm{MnO}{\left(\mathrm{OH}\right)}_{2}+4H^{+}\\ & & \;+{\mathrm{SO}}_{4}^{2-}+2e^{-} \end{array}$$

(12) $${\mathrm{Zn}}_{x}\mathrm{MnO}{\left(\mathrm{OH}\right)}_{2}+4H^{+}+2e^{-}\to n^{2+}+{\mathrm{Zn}}^{2+}+3H_{2}O$$

(13) $${\mathrm{MnO}}_{2}+aH^{+}+ae^{-}↔H_{a}{\mathrm{MnO}}_{2}$$

(14) $${\mathrm{MnO}}_{2}+aH^{+}+b{\mathrm{Zn}}^{2+}+\left(a+2b\right)e^{-}↔H_{a}{\mathrm{Zn}}_{b}{\mathrm{MnO}}_{2}$$

Anode:

(15) $$\mathrm{Zn}↔{\mathrm{Zn}}^{2+}+2e^{-}$$

**Figure 7:**
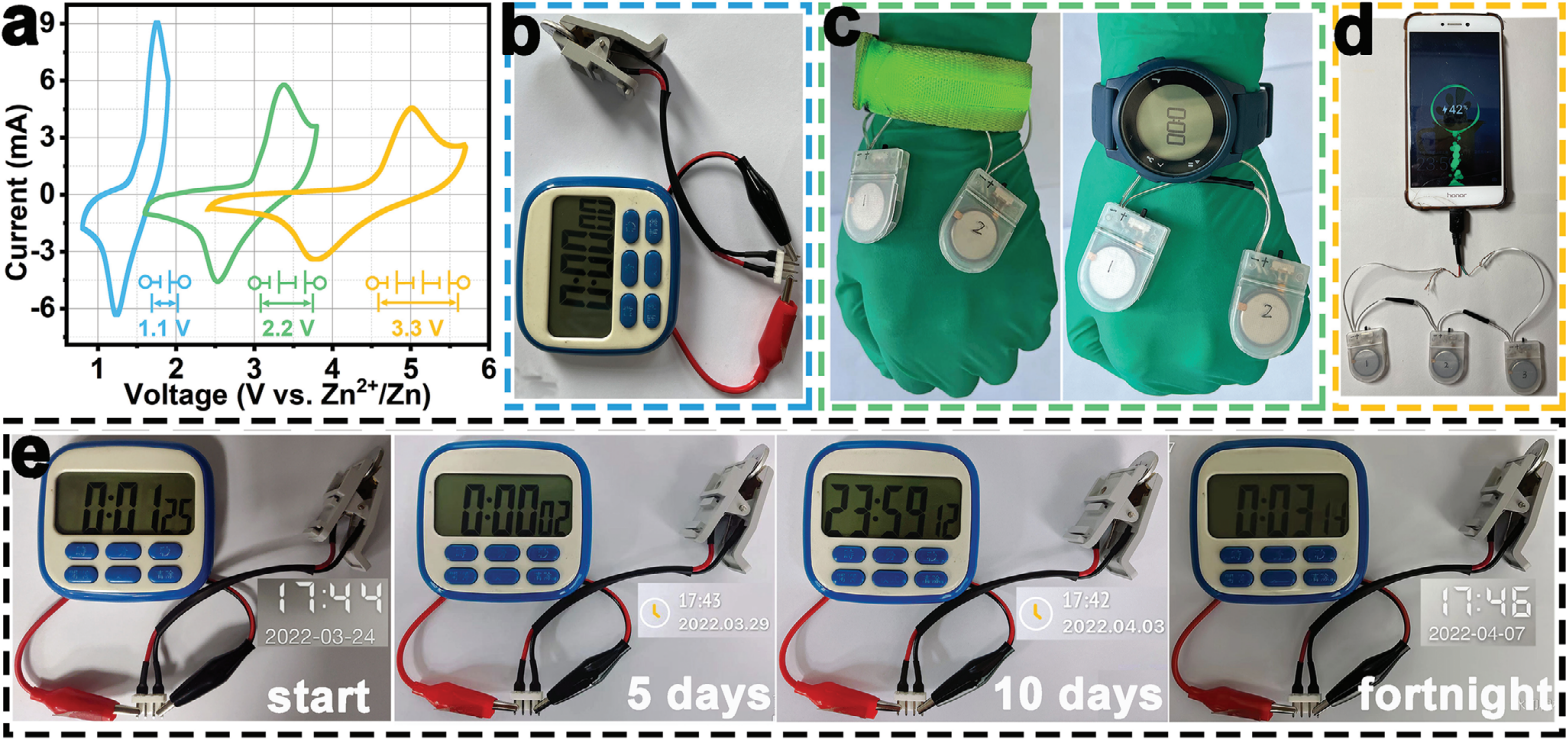
a) CV curves at 10 mV s^−1^ for one, two, and three Zn//a‐MnBO*_x_* units in series. Digital photographs of Zn//a‐MnBO*_x_* cells used to power b) a timer, c) a luminous bracelet and an electronic watch, and d) a mobile phone. e) Digital photographs of a single battery for a lifetime record of timer.

Furthermore, the assembly of many electronic devices verified the practicality of the Zn//a‐MnBO*_x_* batteries. As shown in **Figure** **7**a, the operating voltage range could be further increased by connecting two or three devices in series (each device contains a 1.6 mg mass loading of MnBO*_x_*). It shows that a single battery can drive a timer (Figure 7b), and two batteries in a series can drive light‐emitting bracelets and an electronic watch (Figure 7c), and three batteries in series can even charge a mobile phone (Figure 7d). Finally, the power supply time of the timer was recorded after fully charged to test the actual application time of a single battery. Astonishingly, the timer powered by one battery could be work normally for two weeks (Figure 7e). Therefore, the above results demonstrated the great promise of Zn//a‐MnBO*_x_* battery in widespread application.

## 3 Conclusions

In this work, a‐MnBO*_x_* was successfully synthesized via a simple one‐step coordination method. Detailed in situ and ex situ characterization technologies further revealed the structural evolution of a‐MnBO*_x_* electrodes, proving that three phases of a‐MnO_2_, Zn*_x_* MnO(OH)_2_, and ZHS form on the surface of a‐MnBO*_x_* to generate the heterostructure. Additionally, the newly formed three phases were found to act as the active components for the energy storage. Furthermore, two energy storage modes are involved in the charge‐discharge process for the a‐MnBO*_x_* cathode material, which can be ascribed to the intercalation/deintercalation of H^+^ and Zn^2+^ ions in a‐MnO_2_ phase and reversible conversion mechanism between ZHS and Zn*_x_* MnO(OH)_2_. Meanwhile, the inner a‐MnBO*_x_* with a large specific surface served as a robust framework in the initial energy storage process. Due to the unique physicochemical advantages of a‐MnBO*_x_* cathode material, the constructed Zn//a‐MnBO*_x_* batteries exhibits an excellent energy density of 484.2 Wh kg^−1^ (at a current density of 0.3 A g^−1^), a power density of 12 745.3 W kg^−1^ (at a current density of 20.0 A g^−1^), and an ultra‐long cycling life (specific capacity retention approaches 97.0% after 10 000 cycles). Furthermore, the battery exhibited superior practicability, and one single Zn//a‐MnBO*_x_* battery can drive a timer to work for 14 days. This work provides new opportunities for the further development and grid‐scale application of Zn//Mn‐based batteries.

## Conflict of Interest

The authors declare no conflict of interest.

## Supporting information

Supporting Information

## Data Availability

The data that support the findings of this study are available from the corresponding author upon reasonable request.
