# Supplementary material for: Amorphous Heterostructure Derived from Divalent Manganese Borate for Ultrastable and Ultrafast Aqueous Zinc Ion Storage
Source: Adv Sci (Weinh). 2023 Jan 20;10(8):2205794. doi: 10.1002/advs.202205794 (PMC10015855; doi:10.1002/advs.202205794)
Supplement: Supplementary file 1 — Supporting Information [file ADVS-10-2205794-s001.pdf]

ADVANCED SCIENCE
Open Access

Supporting Information

for *Adv. Sci.*, DOI 10.1002/advs.202205794

Amorphous Heterostructure Derived from Divalent Manganese Borate for Ultrastable and Ultrafast Aqueous Zinc Ion Storage

Xixian Li, Chenchen Ji, Jinke Shen, Jianze Feng, Hongyu Mi*, Yongtai Xu, Fengjiao Guo and Xingbin Yan**

Supporting Information

Amorphous heterostructure derived from divalent manganese borate for ultrastable and ultrafast aqueous zinc ion storage

Xixian Li, Chenchen Ji, Jinke Shen, Jianze Feng, Hongyu Mi*,Yongtai Xu, Fengjiao Guo, and Xingbin Yan**

X. Li, Prof. C. Ji, J. Shen, Prof. H. Mi, Prof. F. Guo,

State Key Laboratory of Chemistry and Utilization of Carbon Based Energy Resources,

School of Chemical Engineering and Technology

Xinjiang University

Urumqi 830017, China

E-mail: jichenchen2010@163.com; mmihongyu@163.com

J. Feng, Y. Xu, Prof. X. Yan

State Key Laboratory of Optoelectronic Materials and Technologies, School of Materials Science and Engineering

Sun Yat-Sen University

Guangzhou, 510275, P. R. China

E-mail: yanxb3@mail.sysu.edu.cn

Prof. C. Ji

State Key Laboratory of Fine Chemicals

Dalian University of Technology

Dalian 116024, China

1. Experimental Section

1.1 Materials

All chemicals were used as received without further purification. Sodium borohydride (98%), zinc sulfate heptahydrate (AR), anhydrous acetonitrile (99.8%, H_2O ≤ 0.005%), Zinc trifluoromethanesulfonate (98%), manganese sulfate monohydrate (AR, 99%) were purchased from Shanghai Aladdin Bio-Chem Technology Co. Ltd. (Shanghai, China). Manganese chloride tetrahydrate (99%) was supplied by Hongyan Chemical Reagent Factory (Tianjin, China). β-MnO_2 (99%) was supplied by Shanghai Macklin Biochemical Co., Ltd. Acetylene black, and polytetrafluoroethylene (PTFE) were purchased from Shenyang Kejing Auto-instrument Co., Ltd., Sigma-Aldrich, respectively. The Whatman GF/D (Glass Microfiber Filters) separators were purchased from Shanghai root biological technology limited company. Graphite paper (thickness: 0.1 mm), and zinc foils (thickness: 0.08 mm) were from commercial supplies.

1.2 Synthesis of amorphous manganese borate (a-$MnBO_x$)

In detail, 0.5 g manganese chloride tetrahydrate ($MnCl_2 \cdot 4H_2O$) and 0.3 g sodium borohydride ($NaBH_4$) powder were respectively added to the round-bottom flask, followed by adding 100 mL deionized water. The reaction flask was placed in an ice bath to carry out the reaction and stirred for

2 hours. The round bottom flask was then removed from the ice bath condition and standed for 10 minutes. After that, the obtained precipitates were washed several times with deionized water, and finally dried at 60°C for 2 h under a vacuum oven.

1.3 Preparation of the positive electrodes

The a-$MnBO_x$ electrodes were fabricated by homogenously mixing the active material powders, acetylene black, and PTFE binder with a mass ratio of 8:1:1. The prepared slurry was mixed uniformly to form a disk electrode (diameter: 12 mm), which was then dried in a vacuum oven overnight. The loading mass of the active material is about 1.6 mg.

1.4 The assembly of the Zn//a-$MnBO_x$ battery

The Zn//a-$MnBO_x$ battery was assembled in the CR2032-type coin cell in the open-air environment, in which the a-$MnBO_x$ electrode was used as the cathode, graphite paper was used as the current collector, Zn foil was used as the anode, 1 M $ZnSO_4$ with 0.1 M $MnSO_4$ aqueous solution were used as the electrolyte and Whatman GF/D (glass microfiber filters) was used as the separator. In addition, the amount of electrolyte was 120 μL in all cases for all the cells.

1.5 Electrochemical measurements

Galvanostatic charge/discharge (GCD) and cycling stability tests were conducted on a Land Battery Test System, and the current densities ranged from 0.3 to 20.0 A g^{-1}. Cyclic voltammetry (CV) tests were measured on an electrochemical workstation (CHI660E, shanghai Chenhua) between 0.8 to 1.9 V under different scan rates changed from 0.1 to 50 mV s^{-1}. The electrochemical impedance spectroscopy (EIS) was carried out at a frequency ranging from 0.01 to 10^5 Hz at an open circuit potential with an amplitude of 5 mV. The calculation of current density and specific capacity was based on the mass of the cathode active material.

The specific energy density (E) and specific capacity (C) were read directly from the Land Battery Test System. The specific power density (P) of the cells was obtained from the following equations:

$$P = \frac{3600E}{\Delta t} \quad \text{(S1)}$$

where E (Wh kg^{-1}) is the energy density, C (mAh g^{-1}) is the specific capacity, P (W kg^{-1}) is the specific power density, and Δt (h) is the discharging time.

1.6 Material Characterizations

The structure of the prepared sample was examined by the X-ray powder diffraction (XRD, Brüker D8) measurement with Cu Kα radiation. Of note, the range of *in-situ* XRD testing was 10-30° with a scan rate of 4°/min. Scanning electron microscopy (SEM, SU-8010), transmission electron microscopy (TEM, FEI Tecnai G2 F30), and high-resolution transmission electron microscope (HRTEM) were employed to reveal the microstructure of the prepared samples. Elemental mapping images were collected from energy dispersive X-ray (EDX) spectroscopy. X-ray photoelectron spectroscopy (XPS, ESCALAB 250X_i) spectra were employed to investigate the surface chemical species of pristine samples and calibrated to the C 1s peak binding energy of 284.8 eV. Furthermore, in the XPS etching test, argon ions were used for etching, and the etching depth was 20 nm. Of note, *ex-situ* tests including XRD, FESEM, HRTEM, and XPS were utilized to analyze the reaction mechanism of the a-$MnBO_x$ cathode at the selected states of the 5th discharge/charge cycle. Raman spectra were performed from a Micro-Raman spectrometer (Brüker Senterra spectrometer). To investigate the water content, Thermogravimetric analysis (TGA) (NETZSCH STA 449F3) was performed from room temperature to 1100°C at a ramping rate of 20°C min^{-1} in the nitrogen atmosphere. Nitrogen adsorption/desorption isotherms were measured on a Micromeritics ASAP 2460 3.00 volumetric adsorption analyzer at 77.3 K.

1.7 Density functional theory (DFT) calculations

To simulate the formation energy on β-MnO_2 and MnB_4O_7, Density functional theory (DFT) calculations were performed using the Vienna Ab initio Simulation Package (VASP) [S1,S2] based on the pseudopotential plane wave (PPW) method. The perdew-Bueke-Ernzerhof (PBE) functional [S3] was used to describe the exchange-correlation effects of electrons. We have chosen the projected augmented wave (PAW) potentials [S4,S5] to describe the ionic cores and take valence electrons into account using a plane wave basis set with a kinetic energy cutoff of 500 eV. To simulate the surface of β-MnO_2 and MnB_4O_7, slab models were built by slicing the (010) and (021) planes for β-MnO_2 and MnB_4O_7, respectively. An extra vacuum zone of 15 A was added to both models to avoid possible interaction. The structures were first relaxed until the force on every atom was less than 0.02 eV/A. The formation energy was calculated by the following equation:

$$E_{\mathrm{f}} = E(\mathrm{A_x B_y}) - \mathrm{x}E(\mathrm{A}) - \mathrm{y}E(\mathrm{B}) \quad \text{(S2)}$$

Where E(A) and E(B) represent the energy of elemental material while $E(A_xB_y)$ represents the energy of the compound.

2. Supplementary Figures and Tables

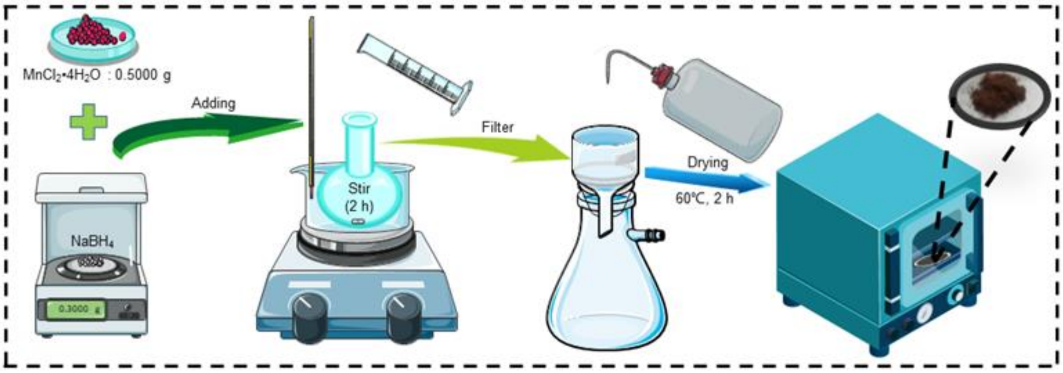

Figure S1. Schematic diagram of the preparation for the a-$MnBO_x$ sample.

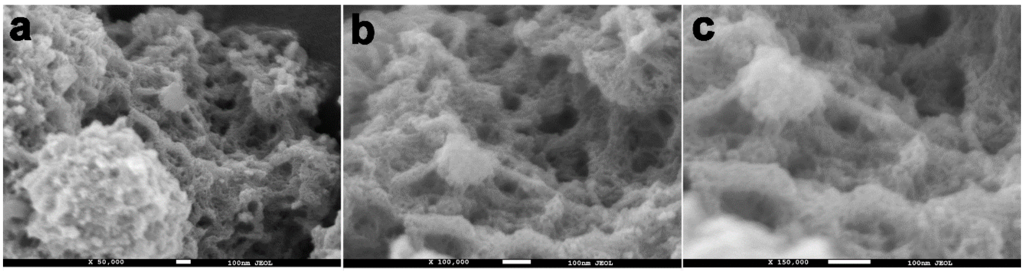

Figure S2. SEM images of a-$MnBO_x$ with different magnifications (scale bar: 100 nm).

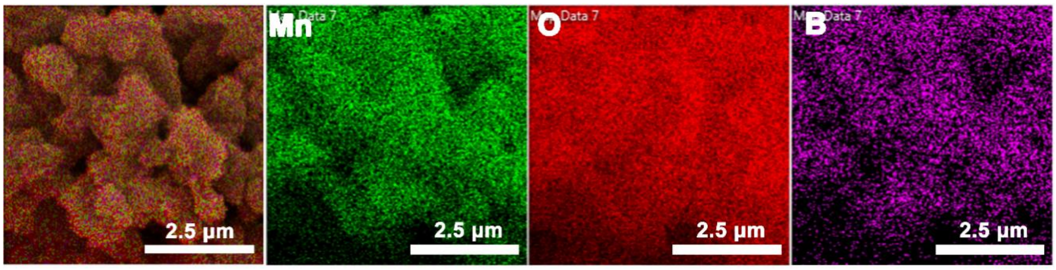

Figure S3. SEM image and the corresponding elemental mapping images of the a-$MnBO_x$ sample.

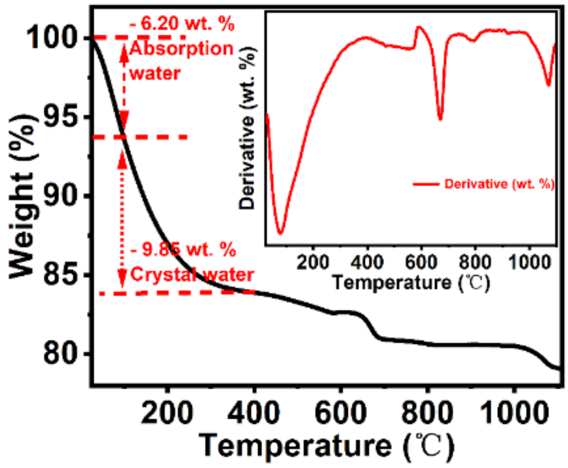

Figure S4. TGA analysis of the a-$MnBO_x$ sample.

The TGA analysis of the a-$MnBO_x$ sample was investigated in the N_2 atmosphere from room temperature to 1100℃, respectively. It can be observed that the weight loss of a-$MnBO_x$ at 400°C is 16.05%. While there is about 6.20% and 9.85% weight loss up to 100℃ and 100 – 400℃ for a-$MnBO_x$, which are attributed to the loss of the physically absorbed water and crystal water within the a-$MnBO_x$ sample, respectively.

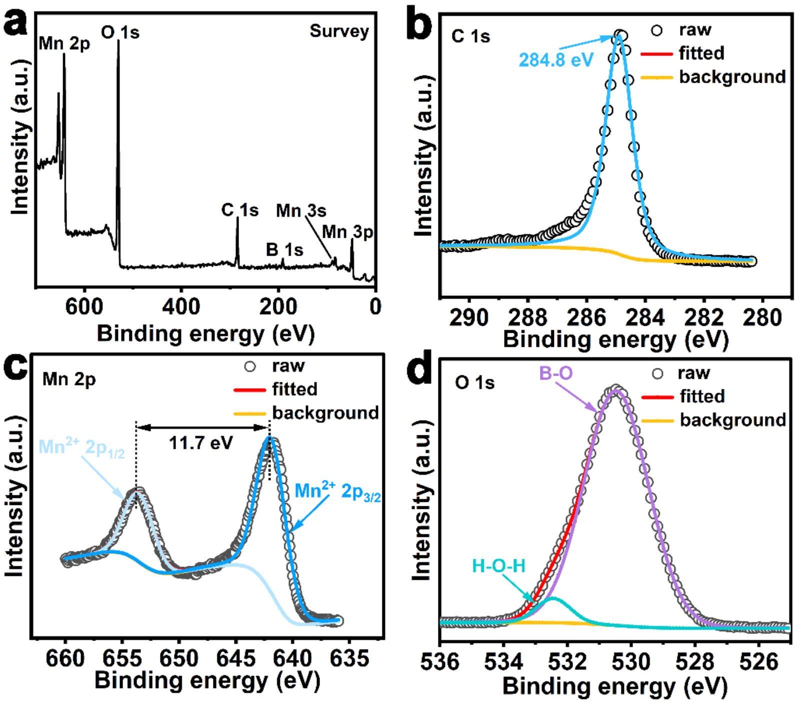

Figure S5. XPS spectra of the a-$MnBO_x$ sample. (a) XPS wide spectrum. (b) C 1s spectrum. (c) Mn 2p spectrum. (d) O 1s spectrum.

All the XPS tests were calibrated form C 1s, and the peak position of the calibrated C1s was 284.8 eV (Figure S5b). From the XPS wide spectrum in Figure S5a, the positions of peaks for the Mn, C, B, and O elements are obtained after calibrating the position peak of C1s. The Mn 2p XPS spectrum (Figure S5c) shows two main peaks at around 653.8 and 642.1 eV with a spin energy separation of 11.7 eV, which are typical of Mn^{2+} $2p_{1/2}$ and Mn^{2+} $2p_{3/2}$ of a-$MnBO_x$ sample, respectively. The high-resolution XPS spectrum of O 1s (Figure S5c) can be deconvoluted into two peaks with binding energies at 530.6 and 532.8 eV, Corresponding to the O-B bond in tetraborate and the H-O-H bond inbound water, respectively.

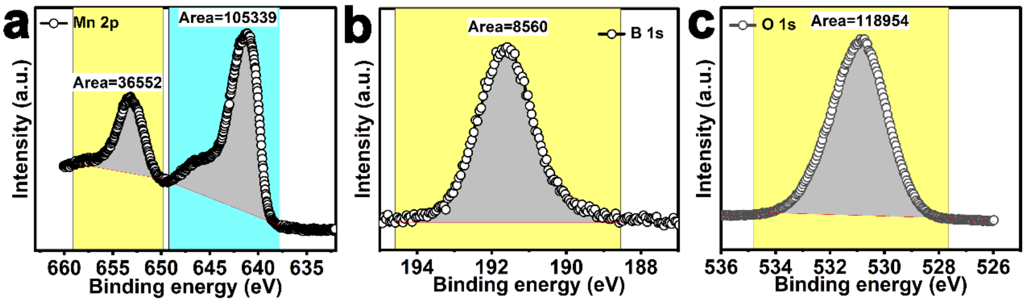

Figure S6. XPS integration of the a-$MnBO_x$ samples. (a) Mn 2p spectrum. (c) B 1s spectrum. (d) O 1s spectrum.

The area integration of the fine XPS spectra of Mn 2p, B 1s, and O 1s was carried out respectively. The relative area obtained was divided by the respective sensitivity factors, and therefore, the obtained relative atoms ratio is Mn: B: O = 1 : 1: 2.86. Combined with the TGA curve, the average chemical composition can be expresses as $MnBO_2·0.86H_2O$.

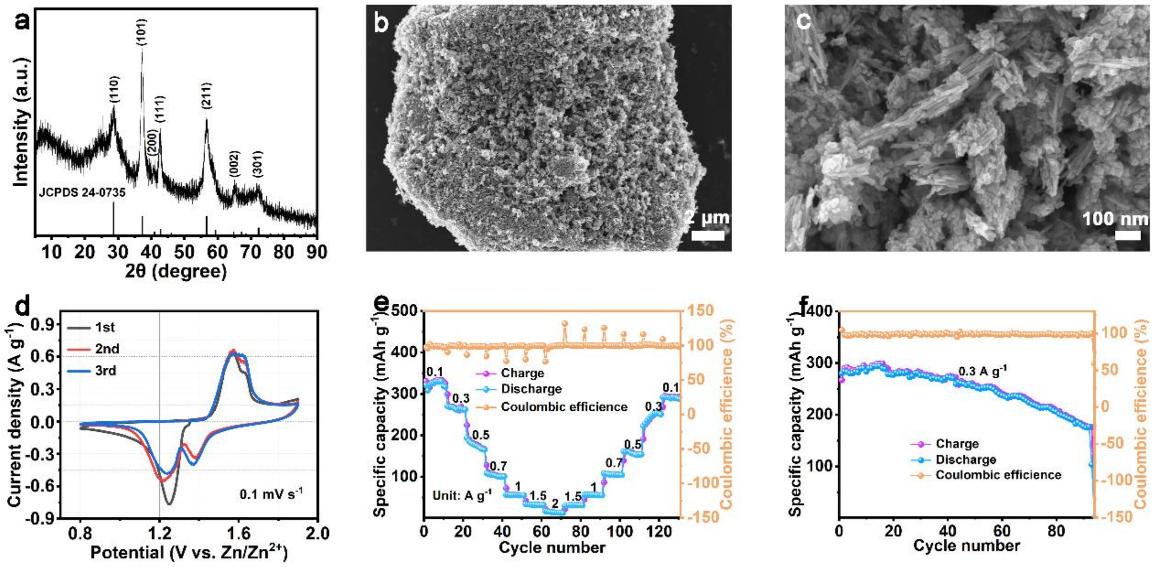

Figure S7. Structural analysis and electrochemical properties of the β-MnO_2. (a) XRD patterns. (b-c) SEM images of β-MnO_2 at low and high magnification. (d) CV curves of the initial 3 cycles of the β-MnO_2 electrode at a scan rate of 0.1 mV s^{-1}. (e) Rate performance of the β-MnO_2//Zn cell. (f) Long cycle performance of the β-MnO_2//Zn battery at 0.3 A g^{-1}.

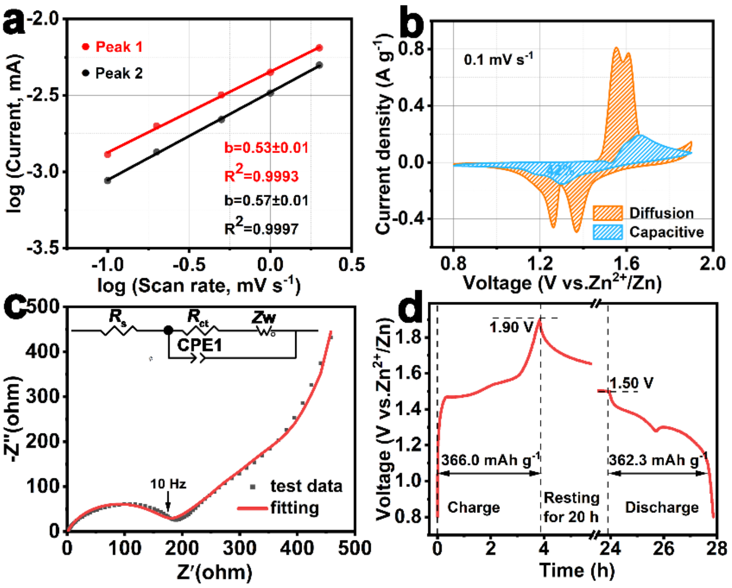

Figure S8. (a) The log (*i*, peak current) vs. log (*v*, scan rate) plots of two peaks in the CV curves of the a-$MnBO_x$ electrode. (b) Diffusion and capacitive contributions at 0.1 mV s^{-1}. (c) EIS after activation of the aqueous Zn//a-$MnBO_x$ battery (the inset shows the equivalent circuit). (d) Self-discharge curve of standing for 20 h in a fully charged state.

The calculation of the *b* value is mainly through the following formula[S6, S7]:

$$i = av^{b} \tag{S3}$$

$$\log(i) = \log(a) + b\log(v) \tag{S4}$$

where *i* is the peak current, *v* is the sweep speed, *a* and *b* are constants.

The area ratio of diffusion control and capacitive-controlled capacitance is calculated by the following Formula S5:

$$i = k_1 v + k_2 v^{\frac{1}{2}} \tag{S5}$$

where k_1v represents the capacitive contribution to the total current and $k_2v^{1/2}$ is the diffusion control current. As shown in Figure S8a, the *b* value of the reduction peak is 0.53, and the *b* value of the corresponding oxidation peak is 0.57. It indicates a solid diffusion-controlled kinetic of a-$MnBO_x$ during the charge/discharge process. At 0.1 mV s^{-1}, the capacitive contribution is 42% in Figure S8b.

As a conclusion, the diffusion contribution is 57.9%, 56.6%, 43.5%, and 29.8% at 0.1, 0.2, 0.5, and 1.0 mV s^{-1}, respectively.

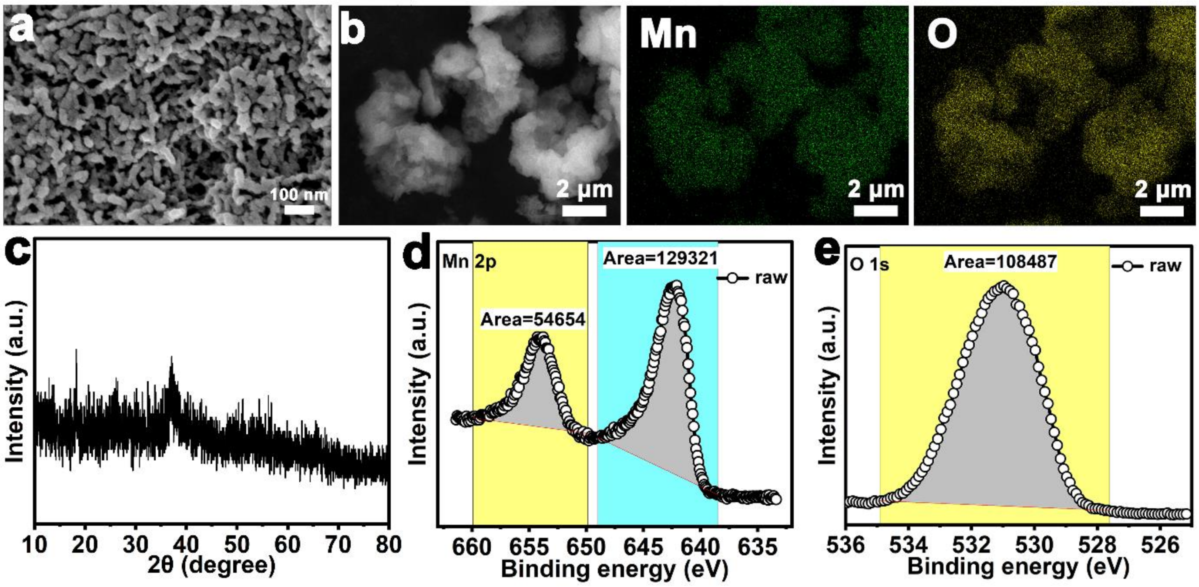

Figure S9. (a) SEM image of a-MnO_2 at high magnification. (b) SEM image of a-MnO_2 alongside its EDS elemental mapping images. (c) XRD pattern. (d) Mn 2p spectrum and corresponding integrated areas. (e) O 1s spectrum and corresponding integrated areas.

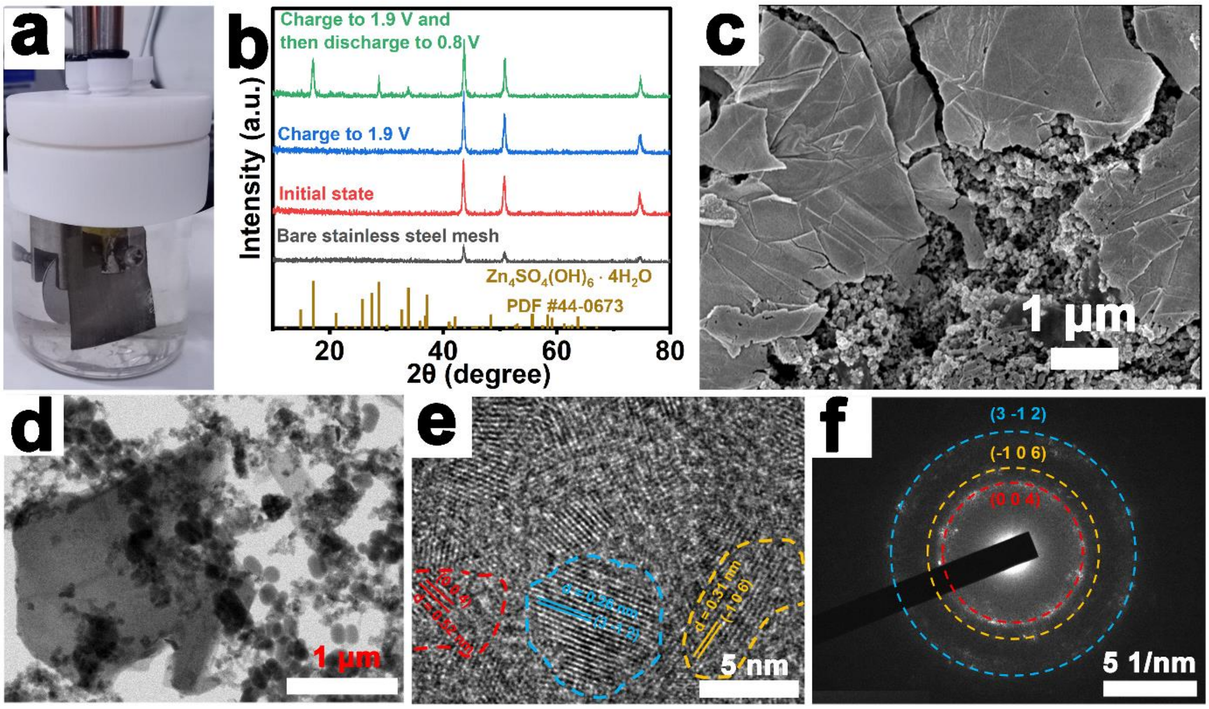

Figure S10. (a) Digital photo of the Zn//a-MnBO$_x$ system. (b) XRD patterns of cathode at different states. (c) SEM image of the cathode material charged to 1.9V and then discharged to 0.8V. (d) TEM image, (e) HRTEM image, and SAED pattern of the cathode material charged to 1.9V and then discharged to 0.8V.

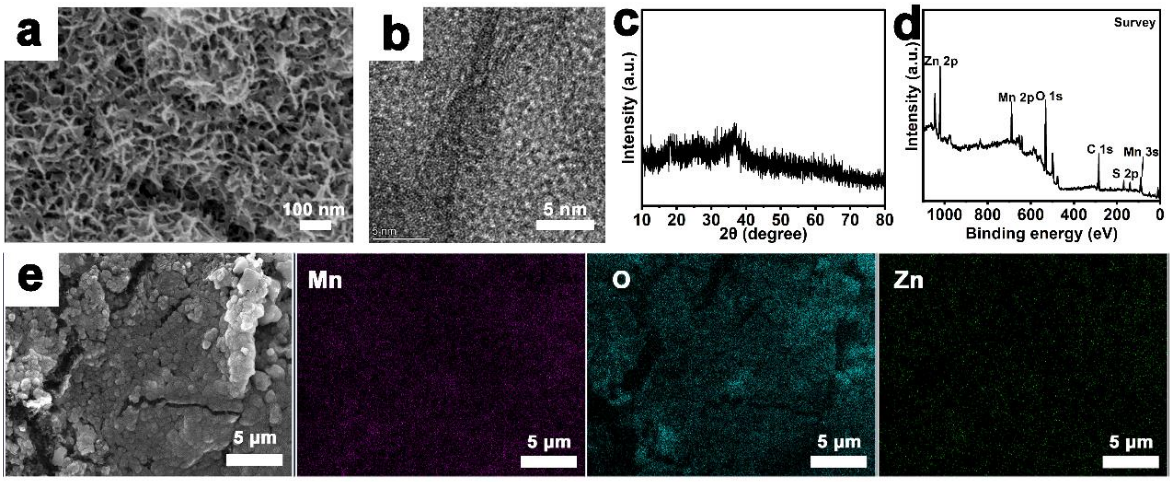

Figure S11. (a) SEM image of Zn$_x$MnO(OH)$_2$ at high magnification. (b) HRTEM image of the Zn$_x$MnO(OH)$_2$. (c) XRD pattern. (d) XPS wide spectrum. (e) SEM image of the Zn$_x$MnO(OH)$_2$ alongside its EDS elemental mapping images.

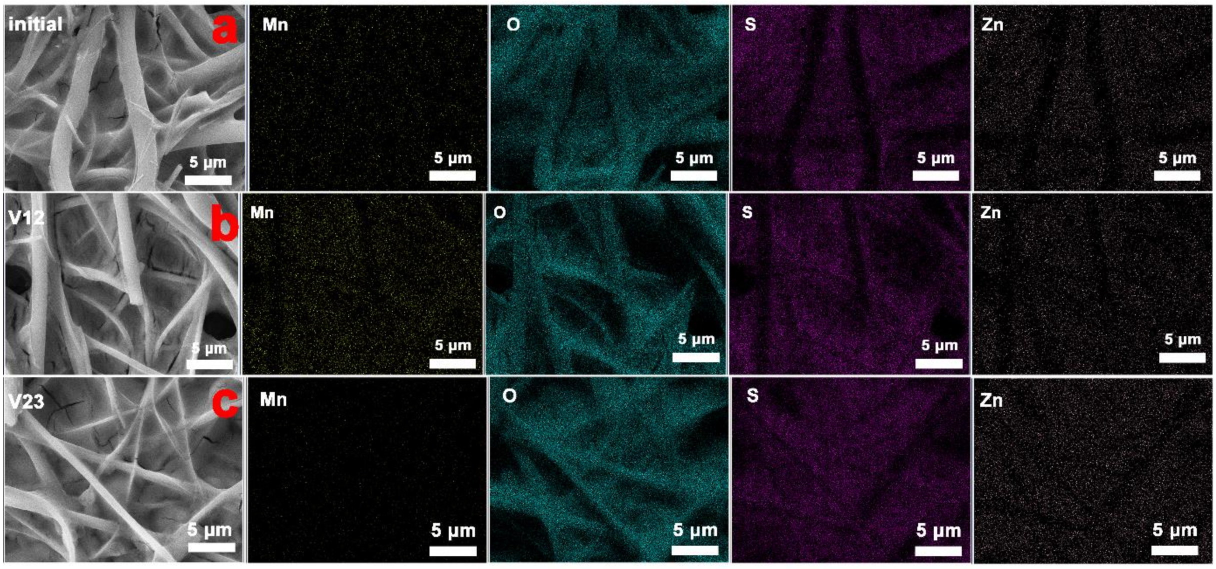

Figure S12. SEM images and the corresponding elemental mapping images (Mn, O, Zn, and S) of the a-MnBO$_x$ electrode at different charge-discharge states (a) initial state, (b) discharged to 0.8 V, and (c) discharged to 0.8 V and then charged to 1.9 V.

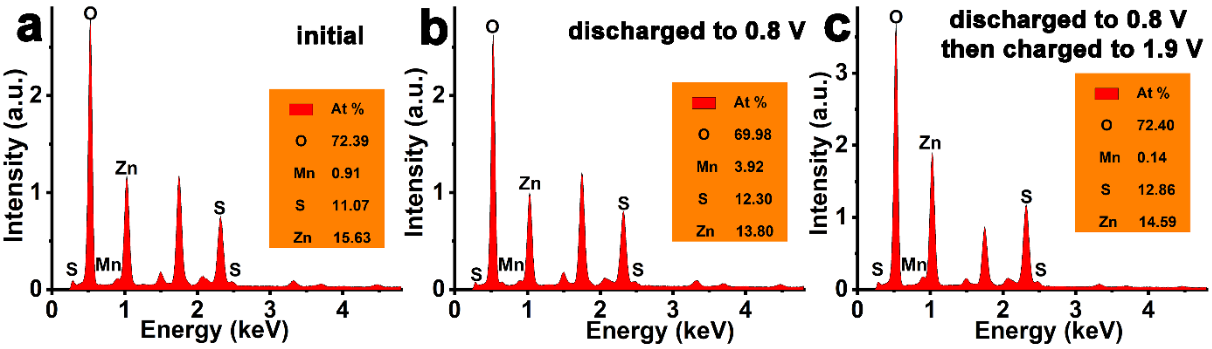

Figure S13. SEM-EDS element analysis of the a-$MnBO_x$ electrode at different charge-discharge states (a) initial state, (b) discharged to 0.8 V, and (c) discharged to 0.8 V and then charged to 1.9 V.

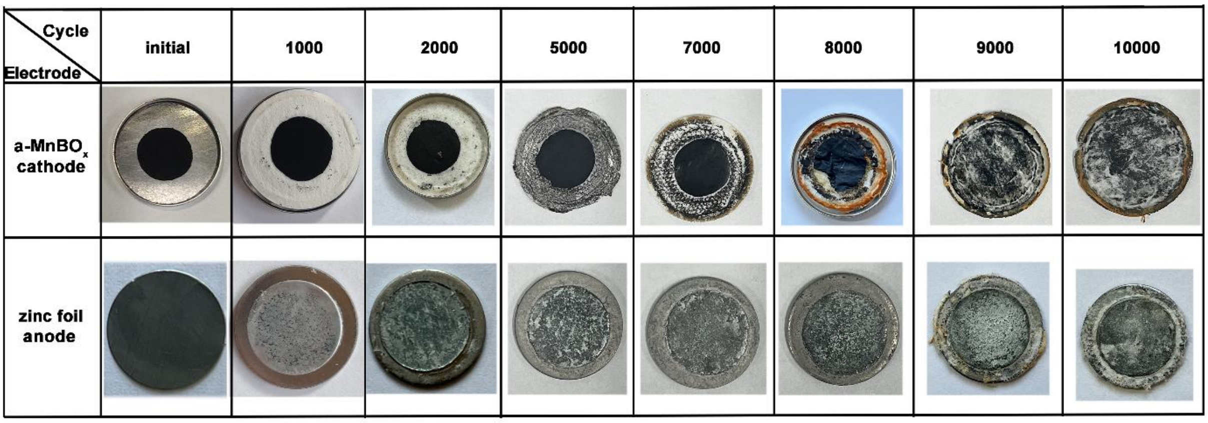

Figure S14. Photographs of the a-$MnBO_x$ cathode and zinc foil anode at different cycles under a current density of 20.0 A g^{-1}.

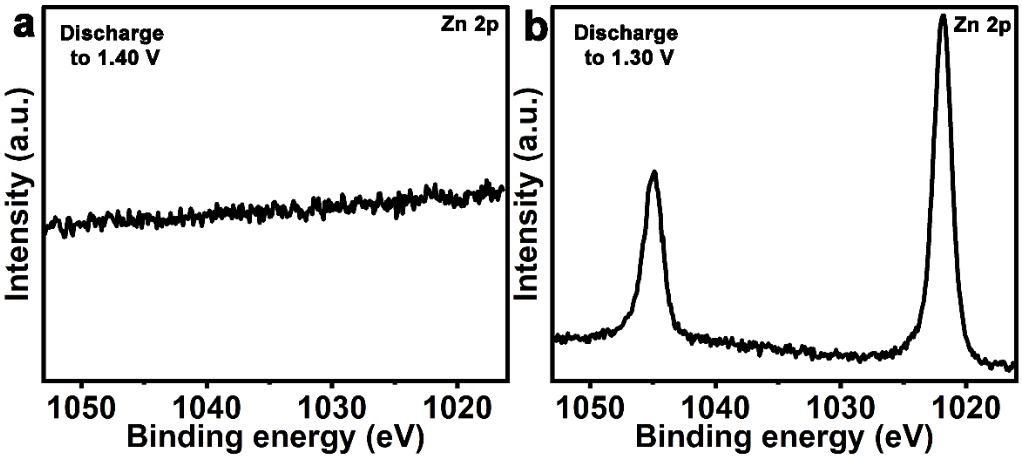

Figure S15. XPS spectra of Zn 2p of the a-$MnBO_x$ electrodes at different discharge states. (a) Discharge to 1.40 V. (b) Discharge to 1.30 V.

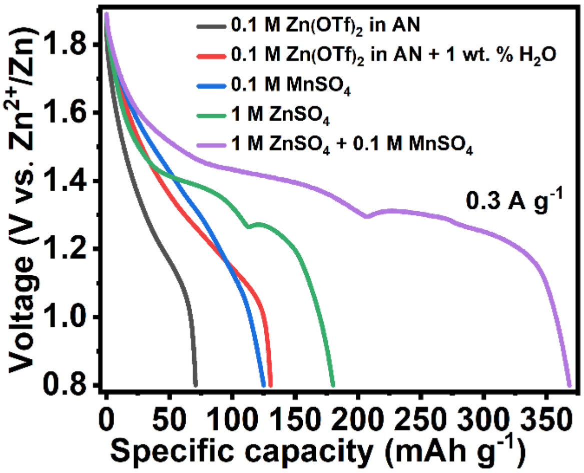

Figure S16. The discharge curves of the a-MnBO$_x$ electrodes tested in different electrolytes at 0.3 A g^{-1} (1 M $ZnSO_4$ + 0.1 M $MnSO_4$, 1 M $ZnSO_4$, 1 M $ZnSO_4$, 0.1 M $Zn(OTf)_2$ in AN, and 0.1 M $Zn(OTF)_2$ in AN+1 wt.% H_2O, respectively).

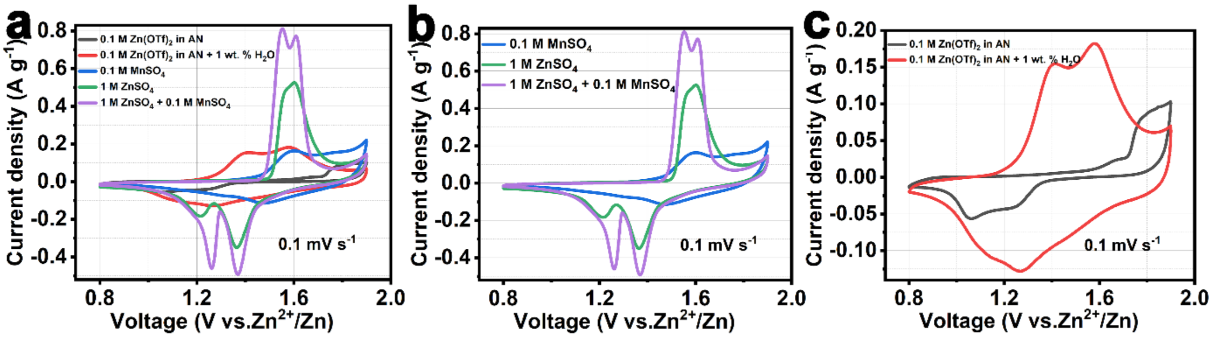

Figure S17. CV curves of the a-MnBO$_x$ electrodes tested in different electrolytes at 0.1 mV s^{-1}. (a) CV curves tested in five electrolytes (1 M $ZnSO_4$ + 0.1 M $MnSO_4$, 1 M $ZnSO_4$, 1 M $ZnSO_4$, 0.1 M $Zn(OTf)_2$ in AN, and 0.1 M $Zn(OTf)_2$ in AN+1 wt.% H_2O, respectively). (b) CV curves tested in 1 M $ZnSO_4$ + 0.1 M $MnSO_4$, 1 M $ZnSO_4$, and 1 M $ZnSO_4$ electrolytes, respectively. (c) CV curves tested in two organic electrolytes (0.1 M $Zn(OTf)_2$ in AN, and 0.1 M $Zn(OTf)_2$ in AN+1 wt.% H_2O, respectively).

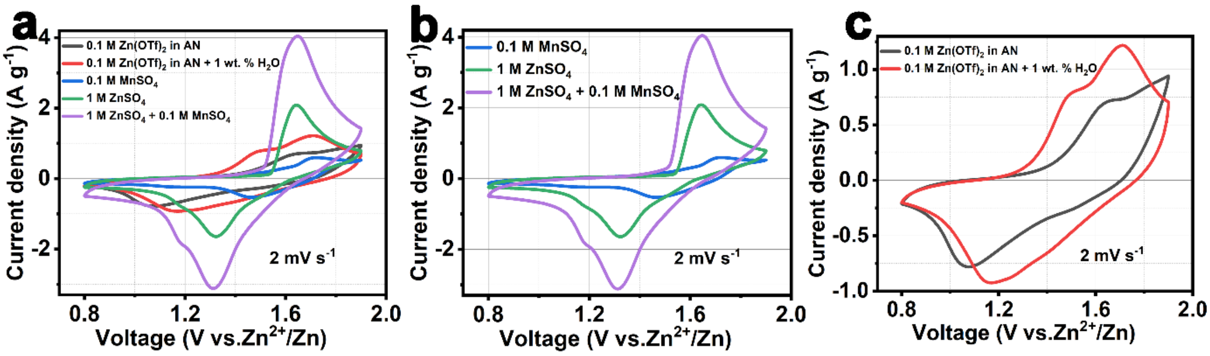

Figure S18. CV curves of the a-MnBO$_x$ electrodes tested in different electrolytes at 2.0 mV s^{-1}. (a) CV curves of the a-MnBO$_x$ electrodes tested in five electrolytes (1 M $ZnSO_4$ + 0.1 M $MnSO_4$, 1 M $ZnSO_4$, 1 M $ZnSO_4$, 0.1 M $Zn(OTf)_2$ in AN, and 0.1 M $Zn(OTf)_2$ in AN+1 wt.% H_2O, respectively). (b) CV curves of

the a-MnBO$_x$ electrodes tested in three aqueous electrolytes (1 M $ZnSO_4$ + 0.1 M $MnSO_4$, 1 M $ZnSO_4$, and 1 M $ZnSO_4$, respectively). (c) CV curves of the a-MnBO$_x$ electrodes tested in two organic electrolytes (0.1 M Zn(OTf)$_2$ in AN, and 0.1 M Zn(OTf)$_2$ in AN+1 wt.% H_2O, respectively).

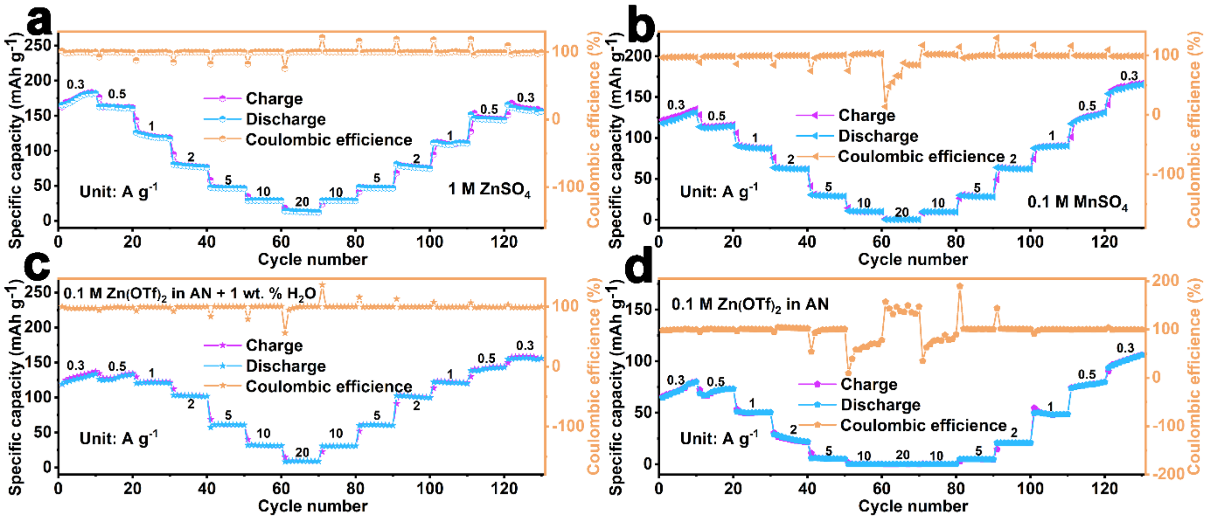

Figure S19. The rate performance of the a-MnBO$_x$ electrodes tested in different electrolytes at different current densities from 0.3 to 20.0 A g^{-1}. (a) 1 M $ZnSO_4$, (b) 0.1 M $MnSO_4$, (c) 0.1 M Zn(OTf)$_2$ in AN+1wt.% H_2O, (d) 0.1 M Zn(OTf)$_2$ in AN.

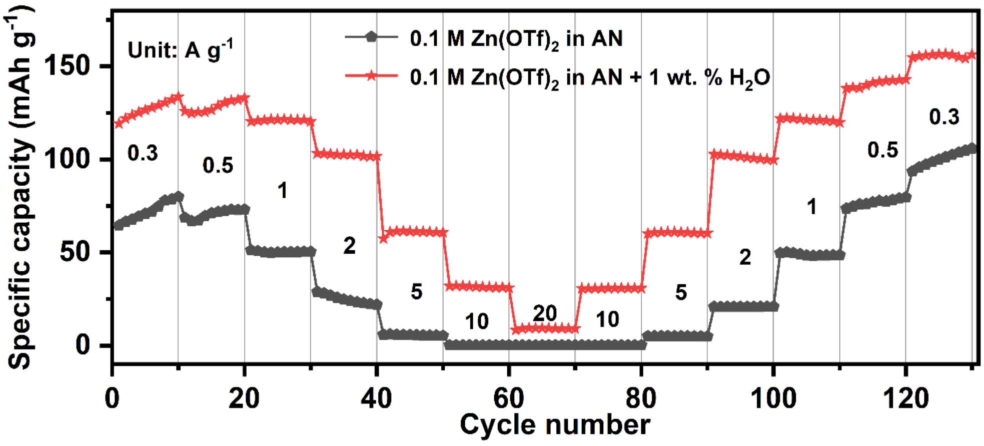

Figure S20. The rate performance of the a-MnBO$_x$ electrode in two organic electrolytes (0.1 M Zn(OTf)$_2$ in AN, and 0.1 M Zn(OTf)$_2$ in AN+1wt.% H_2O, respectively) at different current densities from 0.3 to 20 A g^{-1}.

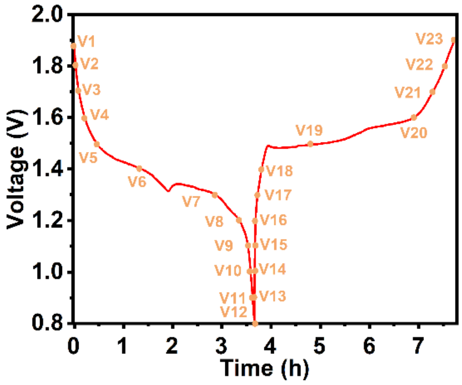

Figure S21. Detailed annotation of the a-$MnBO_x$ cathode at different stages of the charge-discharge curve (applicable to all *ex-situ* tests in this work).

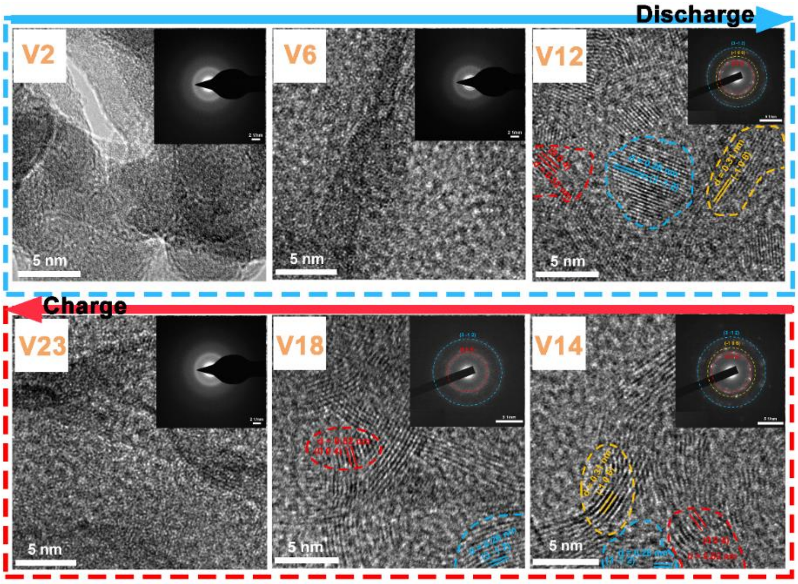

Figure S22. HRTEM images of the a-$MnBO_x$ cathode at different charge and discharge states (at the point of V2, V6, V12, V14, V18, and V19, respectively).

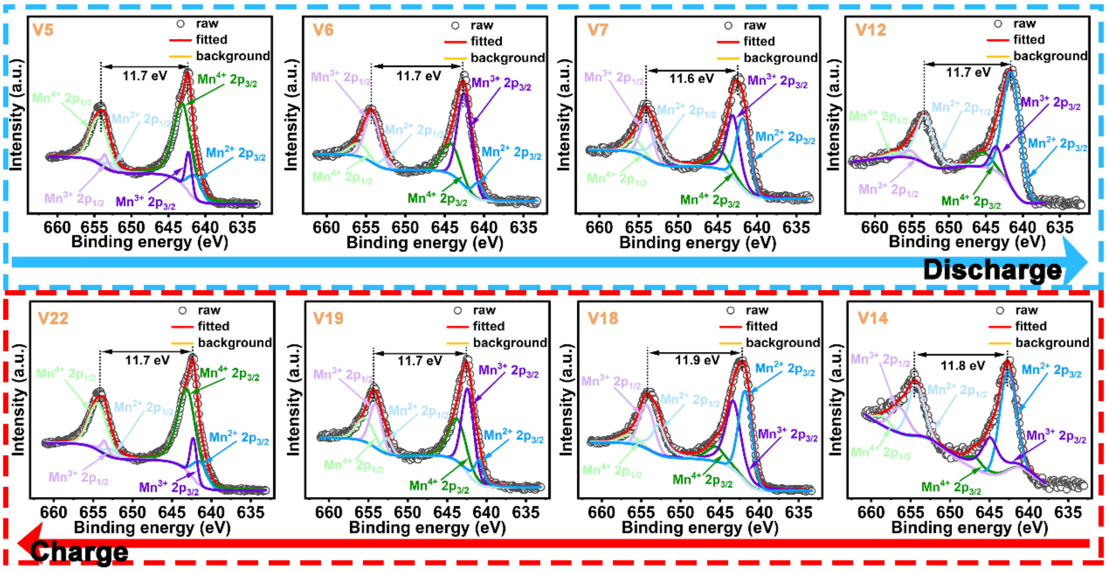

Figure S23. *Ex-situ* XPS analysis of Mn 2p in a-$MnBO_x$ electrode at different charge and discharge states.

status / XPS spectra	Discharge to 1.5 V	Discharge to 1.4 V	Discharge to 1.3 V	Charge to 1.5 V	Charge to 1.8 V
O 1s	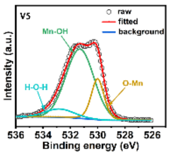	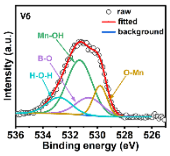	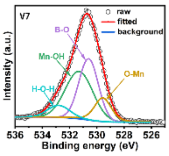	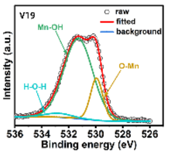	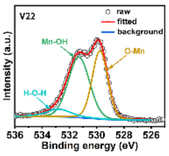
Zn 2p	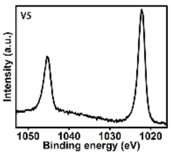	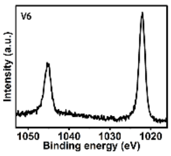	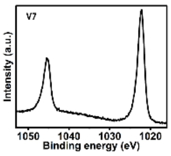	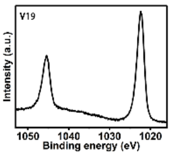	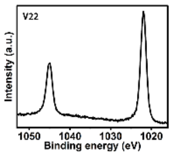

Figure S24. *Ex-situ* XPS analysis of O 1s and Zn 2p in a-$MnBO_x$ electrode sheets at different charge and discharge states.

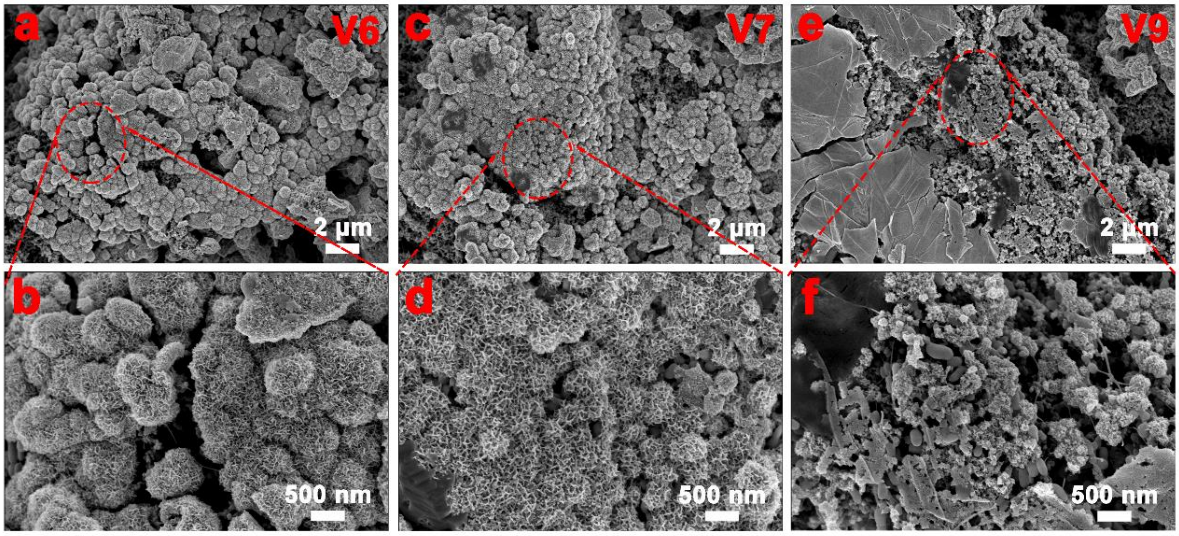

Figure S25. *Ex-situ* SEM images of the a-$MnBO_x$ electrode at different discharge states (a-b) discharged to 1.4 V (V6), (c-d) discharged to 1.3 V (V7), and (e-f) discharged to 1.1 V (V9).

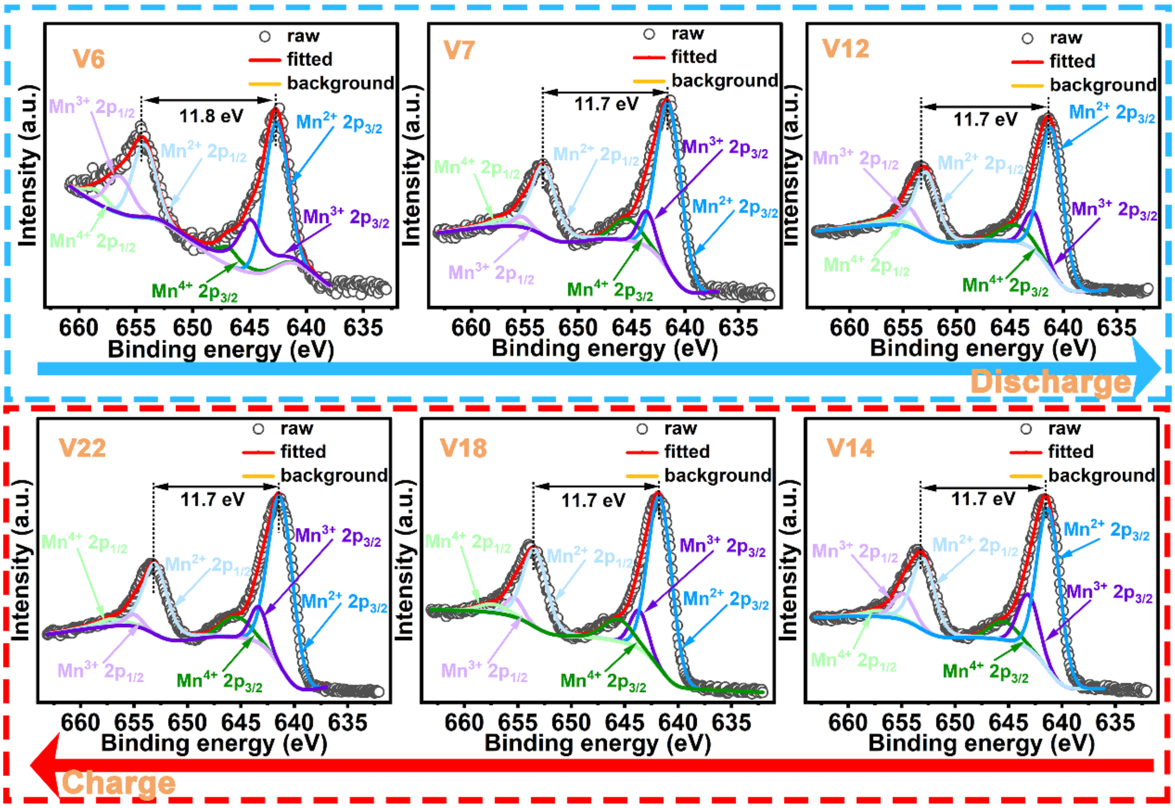

Figure S26. *Ex-situ* XPS analysis of Mn 2p after etching of the a-$MnBO_x$ electrode sheets under different charge and discharge states.

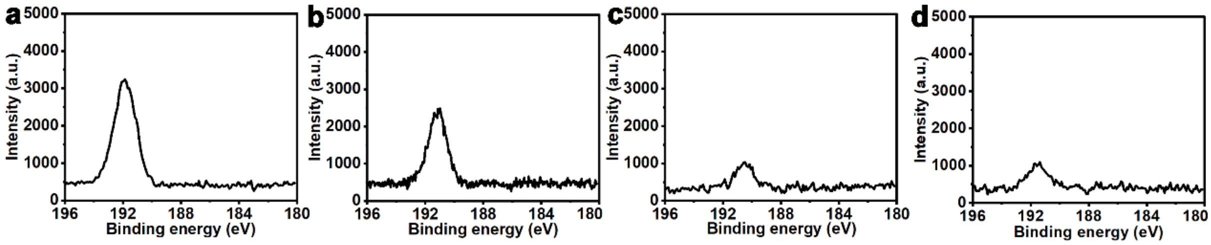

Figure S27. High resolution XPS spectra of the B regions at the (a) initial state, (b) after 5000 cycles, (c) after cell failure, and (d) after cell failure and etched.

The fine XPS spectra of B were tested for the a-$MnBO_x$ electrode in different states. As shown in Figure S27a, the content of element B in the electrode is relatively high in the initial state, which gradually decreases with the continuous charging and discharging (Figure S27b). Finally, when the battery fails, the content of element B in the outer layer and the inner layer is relatively small, which indicates that the electrode will gradually dissolve in the process of charging and discharging (Figure S27c-d).

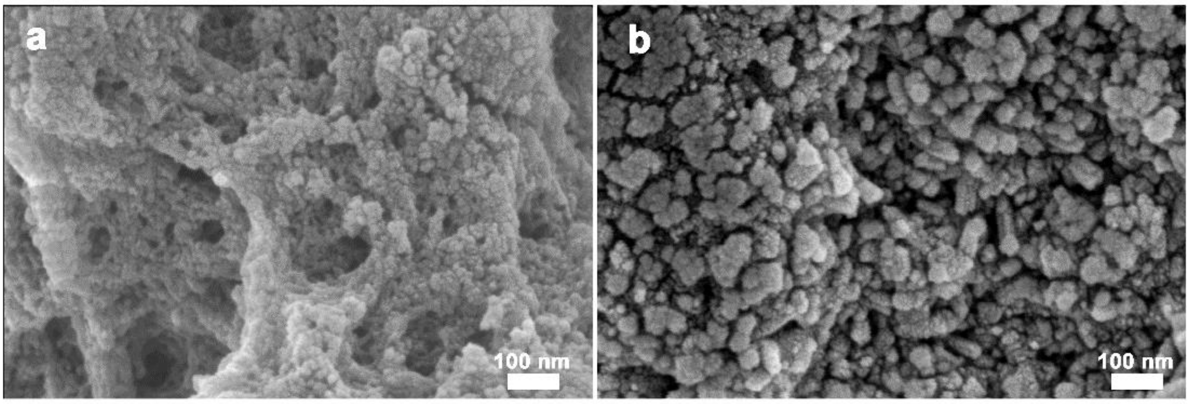

Figure S28. SEM images of the a-$MnBO_x$ electrodes (a) before and (b) after 10,000 cycles at a current density of 20.0 A g^{-1}.

At the current density of 20A g^{-1}, SEM tests were conducted on the a-$MnBO_x$ electrode sheets before and after the battery failure. The surface of the material is loose and porous before cycling tests, but there is a lot of agglomeration after the battery failure (Figure S28).

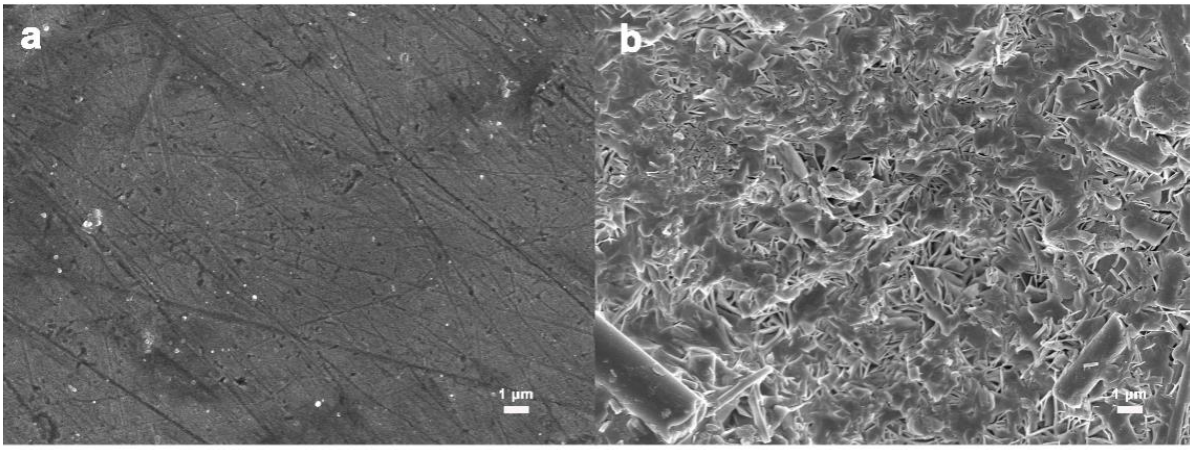

Figure S29. SEM images of the Zn foil anode (a) before and (b) after 10,000 cycles at a current density of 20.0 A g^{-1}.

At the current density of 20A g^{-1}, SEM images show of the Zn foil anode before and after 10,000 cycles. The surface of zinc foil is smooth before cycling, but the surface is covered by a large amount of zinc dendrites after cycling (Figure S29).

Table S1. The pH value of the different electrolytes.

Electrolyte	pH value
1M $ZnSO_4$ +0.1 M $MnSO_4$	4.27
1M $ZnSO_4$	4.40
0.1 M $MnSO_4$	5.73

Table S2. Reaction mechanism of the a-$MnBO_x$ cathode during the charge-discharge process.

Reaction state	Reaction equations
The discharge process	$H_2O \rightarrow H^+ + OH^-$ $MnO_2 + a\ H^+ + a\ e^- \rightarrow H_aMnO_2$ $MnO_2 + a\ H^+ + b\ Zn^{2+} + (a+2b)\ e^- \rightarrow H_aZn_bMnO_2$ $Zn_xMnO(OH)_2 + 4\ H^+ + 2\ e^- \rightarrow Mn^{2+} + Zn^{2+} + 3\ H_2O$ $4\ Zn^{2+} + SO_4^{2-} + 6\ OH^- + 4\ H_2O \rightarrow Zn_4SO_4(OH)_6 \cdot 4H_2O\downarrow$
The charge process	$H^+ + OH^- \rightarrow H_2O$ $H_aMnO_2 \rightarrow MnO_2 + a\ H^+ + a\ e^-$ $H_aZn_bMnO_2 \rightarrow MnO_2 + a\ H^+ + b\ Zn^{2+} + (a+2b)\ e^-$ $Zn_4SO_4(OH)_6 \cdot 4H_2O + Mn^{2+} \rightarrow Zn_xMnO(OH)_2 + 4H^+ + SO_4^{2-} + 2e^-$

Table S3. Summary of the electrochemical performance of typical manganese oxides with different crystal structures and different electrolytes in rechargeable aqueous ZIBs.

Cathode material	Electrolyte	Capacity ($mAh\ g^{-1}$)	Cycle number	Energy density ($Wh\ kg^{-1}$)	Ref.
**a-$MnBO_x$**	**1 M $ZnSO_4$ + 0.1 M $MnSO_4$**	**360.4 (0.3 A g^{-1}) 77.7(20 A g^{-1})**	**10000 (20 A g^{-1}; 97.04%)**	**799**	**This work**
**α-MnO_2**	2 M $ZnSO_4$ + 0.1 M $MnSO_4$	285 (1/3 C)	5000 (5C; 92%)	-	[S6]

**CNT/MnO_2/PEDOT**	2 M $ZnCl_2$ + 0.4 $MnSO_4$	306.1 (1.1 A g^{-1})	2000 (10.8 A g^{-1}; 81.3%)	379.4	[S7]
**$Na_{0.1}MnO_2$· 0.5H_2O**	1 M $ZnSO_4$ (Zn : urea=1:3)	270 (0.3 A g^{-1})	5000 (3 A g^{-1}; 90%)	280	[S8]
**δ-MnO_2**	2 M $ZnSO_4$ + 0.2 M $MnSO_4$	278 (1 C)	10000 (20 C; 66%)	374	[S9]
**MnO_2/CNT**	2 M $ZnSO_4$ + 0.005 M $MnSO_4$	430 （19.5 A g^{-1}）	16000 (19.5 A g^{-1}; nearly 100%)	602	[S10]
**ε-MnO_2**	1 M $ZnSO_4$ + 1 M $MnSO_4$ + 0.1 M H_2SO_4	570 (2 mA cm^{-2})	1800 (30 mA cm^{-2}; 92%)	409 (based on cathode and anode)	[S11]
**$MgMn_2O_4$**	1M $ZnSO_4$ + $MgSO_4$ + 0.1 M $MnSO_4$	269 (50 mA g^{-1})	500 (0.5 A g^{-1}; 80%)	370	[S12]
**$ZnMn_2O_4$/C**	3 M $Zn(CF_3SO_3)_2$	150 (50 mA g^{-1})	500 (0.5 A g^{-1}; 94%)	-	[S13]
**σ-MnO_2 (V_o)**	1 M $ZnSO_4$ + 0.2 M $MnSO_4$	345 (0.2 A g^{-1})	2000 (5 A g^{-1}; 84%)	470	[S14]
**N-MnO_{2-x}@TiC/C**	2 M $ZnSO_4$ + 0.2 M $MnSO_4$	285 (0.2 A g^{-1})	1000 (1 A g^{-1}; 85.7%)	386.5	[S15]

**Na:MnO_2/GCF**	2 M $ZnSO_4$ + 0.1M $MnSO_4$	381.8 (0.1 A g^{-1})	1000 (1 A g^{-1}; 71%)	511.9	[S16]
**Amorphous-MnO_2**	2 M $ZnSO_4$	87 (5 A g^{-1})	5000 (5 A g^{-1}; 93.18%)	-	[S17]
**$Ni_xMn_{3-x}O_4$@C**	2 M $ZnSO_4$ + 0.15 M $MnSO_4$	139.7 (0.05 A g^{-1})	850 (0.4 A g^{-1}; 91.9%)	178.1	[S18]
**P-MnO_{2-x}@VMG**	3 M LiCl + 2 M $ZnCl_2$ + 0.4 M $MnSO_4$ +PVA	302.8 (0.5 A g^{-1})	1000 (2 A g^{-1}; 91.3%)	369.5	[S19]
**δ-MnO_2**	1 M $Zn(TFSI)_2$ + 0.1 M $Mn(TFSI)_2$	238.8 (0.2 C)	4000 (20 C; 93%)	-	[S20]
**$Ca_{0.28}MnO_2\cdot 0.5H_2O$**	1 M $ZnSO_4$ + 0.1 M $MnSO_4$	298 (175 mA g^{-1})	5000 (3.5 A g^{-1})	-	[S21]
**δ-MnO_2**	2 M $ZnSO_4$ + 0.1 M $MnSO_4$ + PVA	219.58 (1.5 mA cm^{-2})	10000 (10 mA cm^{-2})	432.05	[S22]
**β-MnO_2 (V_o)**	3 M $ZnSO_4$ + 0.1 M $MnSO_4$	302 (0.05 A g^{-1})	300 (0.5 A g^{-1}; 94%)	-	[S23]
**PANI-MnO_2**	2 M $ZnSO_4$ + 0.1 M $MnSO_4$	298 (0.2 A g^{-1})	5000 (2 A g^{-1}; 40%)	-	[S24]

**β-MnO_2**	3 M $Zn(CF_3SO_3)_2$ + 0.1 M $Mn(CF_3SO_3)_2$	258 (0.65C)	2000 (6.5 C; 94%)	75.2 (based on whole battery mass)	[S25]
**O_d-MnO_2**	2 M $ZnSO_4$ + 0.1 M $MnSO_4$	290 (0.3 A g^{-1})	1000 (1 A g^{-1}; nearly 100%)	406	[S26]
**$K_{0.8}Mn_8O_{16}$ (V_o)**	2 M $ZnSO_4$ + 0.1 M $MnSO_4$	216 (0.1 A g^{-1})	1000 (1 A g^{-1}; nearly 100%)	398	[S27]
**δ-MnO_2**	2 M $ZnSO_4$ + 0.1 M $MnSO_4$	358 (0.3 A g^{-1})	2000 (3 A g^{-1}; 95.7%)	397	[S28]
**Mn^{2+}/ε-MnO_2 //Zn/$Zn(OH)_4^{2-}$**	0.5 M H_2SO_4 + 1.0 M $MnSO_4$ // 2.4 M KOH + 0.1 M $Zn(CH_3COO)_2$	616 (2 mA cm^{-2})	1500 (2 mA cm^{-2}; 97.5%)	1503	[S29]
**Mn^{2+}/ε-MnO_2 //Zn/$Zn(OH)_4^{2-}$**	3M H_2SO_4 + 0.1 M $MnSO_4$ // 0.1 M K_2SO_4 // 6 M KOH	616 (0.1 A g^{-1})	116 (0.5 A g^{-1}; 97.6%)	1621.7	[S30]
**Zn-doped MnO_2**	2 M $ZnSO_4$ + 0.2 M $MnSO_4$	110 (0.5 A g^{-1})	100 (0.5 A g^{-1};66%)	628	[S31]

References

[S1] G. Kresse, J. Furthmüller, *Comput. Mater. Sci.* **1996**, *6*, 15.

[S2] G. Kresse, Hafner, *J. Phys. Rev. B* **1994**, *49*, 14251.

[S3] J. P. Perdew, K. Burke, M. Ernzerhof, *Phys. Rev. Lett.* **1996**, *77*, 3865.

[S4] P. E. Blöchl, O. Jepsen, O. K. Andersen, *Phys. Rev. B* **1994**, *49*, 16223.

[S5] G. Kresse, D. Joubert, *Phys. Rev. B* **1999**, *59*, 1758.

[S6] H. Pan, Y. Shao, P. Yan, Y. Cheng, K. S. Han, Z. Nie, C. Wang, J. Yang, X. Li, P. Bhattacharya, K. T. Mueller, J. Liu, *Nat. Energy* **2016**, *1*, 16039.

[S7] X. Zhang, S. Wu, S. Deng, W. Wu, Y. Zeng, X. Xia, G. Pan, Y. Tong, X. Lu, *Small Methods* **2019**, *3*, 1900525.

[S8] Z. Hou, M. Dong, Y. Xiong, X. Zhang, H. Ao, M. Liu, Y. Zhu, Y. Qian, *Small* **2020**, *16*, e2001228.

[S9] D. Wang, L. Wang, G. Liang, H. Li, Z. Liu, Z. Tang, J. Liang, C. Zhi, *ACS Nano* **2019**, *13*, 10643.

[S10] X. Shen, X. Wang, Y. Zhou, Y. Shi, L. Zhao, H. Jin, J. Di, Q. Li, *Adv. Funct. Mater.* **2021**, *31*, 2101579.

[S11] D. Chao, W. Zhou, C. Ye, Q. Zhang, Y. Chen, L. Gu, K. Davey, S. Z. Qiao, *Angew. Chem. Int. Ed.* **2019**, *58*, 7823.

[S12] V. Soundharrajan, B. Sambandam, S. Kim, V. Mathew, J. Jo, S. Kim, J. Lee, S. Islam, K. Kim, Y.-K. Sun, J. Kim, *ACS Energy Lett.* **2018**, *3*, 1998.

[S13] N. Zhang, F. Cheng, Y. Liu, Q. Zhao, K. Lei, C. Chen, X. Liu, J. Chen, *J. Am. Chem. Soc.* **2016**, *138*, 12894.

[S14] T. Xiong, Z. G. Yu, H. Wu, Y. Du, Q. Xie, J. Chen, Y. W. Zhang, S. J. Pennycook, W. S. V. Lee, J. Xue, *Adv. Energy Mater.* **2019**, *9*, 1803815.

[S15] Y. Zhang, S. Deng, M. Luo, G. Pan, Y. Zeng, X. Lu, C. Ai, Q. Liu, Q. Xiong, X. Wang, X. Xia, J. Tu, *Small* **2019**, *15*, e1905452.

[S16] Y. Wu, M. Wang, Y. Tao, K. Zhang, M. Cai, Y. Ding, X. Liu, T. Hayat, A. Alsaedi, S. Dai, *Adv. Funct. Mater.* **2019**, *30*, 1907120.

[S17] Z. Shen, Z. Tang, C. Li, L. Luo, J. Pu, Z. Wen, Y. Liu, Y. Ji, J. Xie, L. Wang, Y. Yao, G. Hong, *Adv. Energy Mater*. **2021**, *11*, 2102055.

[S18] J. Long, J. Gu, Z. Yang, J. Mao, J. Hao, Z. Chen, Z. Guo, *J. Mater. Chem. A* **2019**, *7*, 17854.

[S19] Y. Zhang, S. Deng, G. Pan, H. Zhang, B. Liu, X. L. Wang, X. Zheng, Q. Liu, X. Wang, X. Xia, J. Tu, *Small Methods* **2020**, *4*, 1900828.

[S20] Y. Jin, L. Zou, L. Liu, M. H. Engelhard, R. L. Patel, Z. Nie, K. S. Han, Y. Shao, C. Wang, J. Zhu, H. Pan, J. Liu, *Adv. Mater.* **2019**, *31*, e1900567.

[S21] T. Sun, Q. Nian, S. Zheng, J. Shi, Z. Tao, *Small* **2020**, *16*, e2000597.

[S22] Y. Jiang, D. Ba, Y. Li, J. Liu, *Adv. Sci.* **2020**, *7*, 1902795.

[S23] M. Han, J. Huang, S. Liang, L. Shan, X. Xie, Z. Yi, Y. Wang, S. Guo, J. Zhou, *iScience* **2020**, *23*, 100797.

[S24] J. Huang, Z. Wang, M. Hou, X. Dong, Y. Liu, Y. Wang, Y. Xia, *Nat. Commun.* **2018**, *9*, 2906.

[S25] N. Zhang, F. Cheng, J. Liu, L. Wang, X. Long, X. Liu, F. Li, J. Chen, *Nat. Commun.* **2017**, *8*, 405.

[S26] J. Wang, J. G. Wang, X. Qin, Y. Wang, Z. You, H. Liu, M. Shao, *ACS Appl. Mater. Interfaces* **2020**, *12*, 34949.

[S27] G. Fang, C. Zhu, M. Chen, J. Zhou, B. Tang, X. Cao, X. Zheng, A. Pan, S. Liang, *Adv. Funct. Mater.* **2019**, *29*, 1808375.

[S28] J. Wang, J.-G. Wang, H. Liu, C. Wei, F. Kang, *J. Mater. Chem. A* **2019**, *7*, 13727.

[S29] L. Zhang, L. Chen, X. Zhou, Z. Liu, *Adv. Energy Mater.* **2015**, *5*, 1400930.

[S30] C. Zhong, B. Liu, J. Ding, X. Liu, Y. Zhong, Y. Li, C. Sun, X. Han, Y. Deng, N. Zhao, W. Hu, *Nat. Energy* **2020**, *5*, 440.

[S31] W. Zhao, J. Fee, H. Khanna, S. March, N. Nisly, S. Joy B. Rubio, C. Cui, Z.Li and Steven L. Sui, *J. Mater. Chem. A* **2022**, *10*, 6762.
